# Diurnal rhythms of wrist temperature are associated with future disease risk in the UK Biobank

**DOI:** 10.1038/s41467-023-40977-5

**Published:** 2023-08-24

**Authors:** Thomas G. Brooks, Nicholas F. Lahens, Gregory R. Grant, Yvette I. Sheline, Garret A. FitzGerald, Carsten Skarke

**Affiliations:** 1 Institute for Translational Medicine and Therapeutics (ITMAT), University of Pennsylvania Perelman School of Medicine, Philadelphia, PA USA; 2 Department of Genetics, University of Pennsylvania Perelman School of Medicine, Philadelphia, PA USA; 3 Department of Radiology, University of Pennsylvania Perelman School of Medicine, Philadelphia, PA USA; 4 Department of Psychiatry, University of Pennsylvania Perelman School of Medicine, Philadelphia, PA USA; 5 Department of Neurology, University of Pennsylvania Perelman School of Medicine, Philadelphia, PA USA; 6 Department of Medicine, University of Pennsylvania Perelman School of Medicine, Philadelphia, PA USA; 7 Department of Systems Pharmacology and Translational Therapeutics, University of Pennsylvania Perelman School of Medicine, Philadelphia, PA USA

**Keywords:** Predictive markers, Diseases, Signs and symptoms, Epidemiology

## Abstract

Many chronic disease symptomatologies involve desynchronized sleep-wake cycles, indicative of disrupted biorhythms. This can be interrogated using body temperature rhythms, which have circadian as well as sleep-wake behavior/environmental evoked components. Here, we investigated the association of wrist temperature amplitudes with a future onset of disease in the UK Biobank one year after actigraphy. Among 425 disease conditions (range *n* = 200-6728) compared to controls (range *n* = 62,107-91,134), a total of 73 (17%) disease phenotypes were significantly associated with decreased amplitudes of wrist temperature (Benjamini-Hochberg FDR q < 0.05) and 26 (6.1%) PheCODEs passed a more stringent significance level (Bonferroni-correction α < 0.05). A two-standard deviation (1.8° Celsius) lower wrist temperature amplitude corresponded to hazard ratios of 1.91 (1.58-2.31 95% CI) for NAFLD, 1.69 (1.53-1.88) for type 2 diabetes, 1.25 (1.14-1.37) for renal failure, 1.23 (1.17-1.3) for hypertension, and 1.22 (1.11-1.33) for pneumonia (phenome-wide atlas available at http://bioinf.itmat.upenn.edu/biorhythm_atlas/). This work suggests peripheral thermoregulation as a digital biomarker.

## Abstract (web summary)

Many chronic diseases present with desynchronized sleep-wake cycles, indicative of disrupted biorhythms. Here, the authors propose peripheral thermoregulation as a digital biomarker based on the association between lower temperature rhythms measured from wearable activity trackers with future onset of disease, such as type 2 diabetes, non-alcoholic fatty liver disease, hypertension and pneumonia.

## Introduction

The benefits of regular physical activity and sufficient sleep are pillars of public health^1,2^. Efforts to assess this linkage objectively at the population level have utilized accelerometry in a phenome-wide scan to associate physical inactivity with a broad range of chronic diseases in the UK Biobank (UKBB)^3,4^ and the All of Us Research Program^5^. The role biorhythms play as biomarkers for disease onset is less well understood^6^. Although a few disease-specific studies, limited to patients with mood disorders^7,8^, have measured the degree of disrupted rest-activity cycles from accelerometry in the UKBB, a comprehensive phenome-wide approach has not yet been established.

Temperature rhythms are well-established biomarkers for circadian clock function^9^. Peripheral wrist temperature oscillations serve as a proxy for endogenous circadian function—through the thermoregulatory coupling of core and periphery—and run inverse to the core clock^10,11^. These traces retain an endogenous sinusoidal component under conditions of constant temperature and light with standardized food intake^11^ and are therefore suitable to estimate circadian entrainment, comparable to melatonin or core body temperature, as marker rhythms^12,13^. Shift work reduces the amplitude of temperature rhythms^14^ and disruption of temperature rhythms has been strongly associated with metabolic syndrome and diabetes^15^, as well as sleep-disordered breathing^16^.

In this work, we explore the relationship between wrist temperature amplitudes and the future onset of diseases by utilizing temperature data collected through an embedded sensor in the actigraphy device that was used for the UKBB study,^17,18^. Importantly, participants collected these data under real life conditions so that the wrist temperature rhythms contain both circadian as well as sleep-wake behavior and environmentally evoked components (i.e., sleep reduces core temp and increases distal skin temperature)^19^. We find that up to 17% of disease conditions among the phenome-wide scan (73 out of 425) are significantly associated with decreased amplitudes of wrist temperature where a two-standard deviation (1.8° Celsius) lower wrist temperature amplitude corresponds to a 91% increased risk for a future diagnosis for nonalcoholic fatty liver disease (NAFLD), 69% for type 2 diabetes, 25% for renal failure, 23% for hypertension, and 22% for pneumonia. We show the comprehensive phenome-wide atlas of the identified mappings at http://bioinf.itmat.upenn.edu/biorhythm_atlas/. This work strongly suggests peripheral thermoregulation as a digital biomarker.

## Results

### Wrist temperatures are concordant with a biorhythm signal

**Fig. 1:**
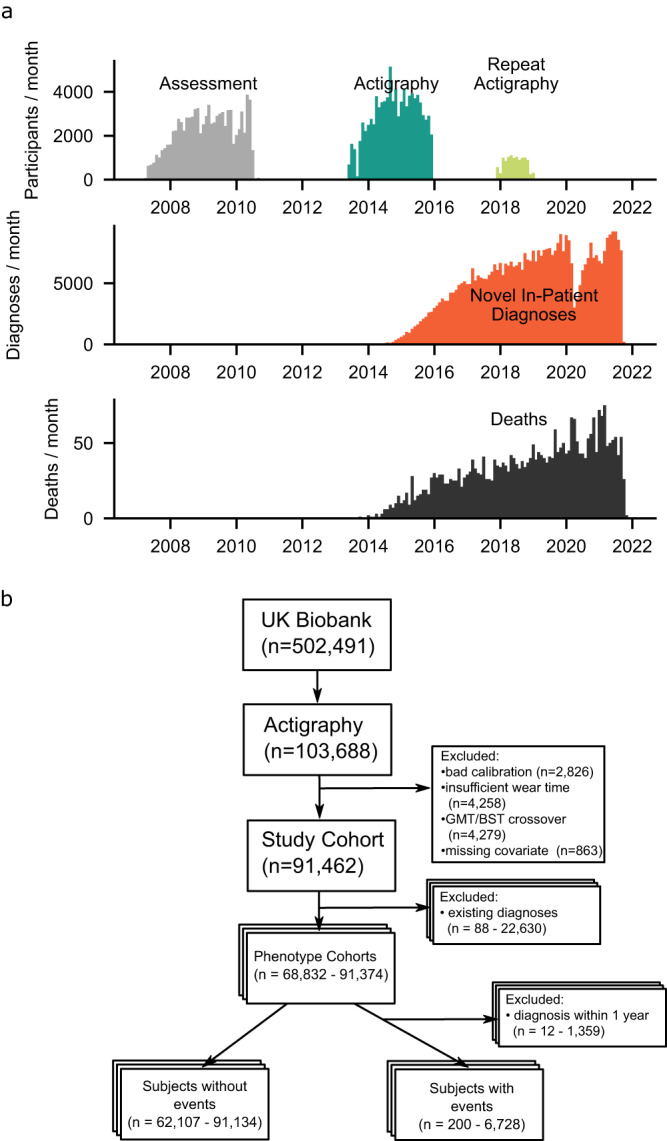
Study design. The UK Biobank actigraphy study yielded 91,462 individuals with high-quality actigraphy health measurements and complete covariate data. A total of 451,994 distinct patient-diagnoses (4.9 per participant) and 3061 deaths (3.3%) were recorded among these participants as per data downloaded on February 23, 2022. **a** Timeline of data collection by year. Participants had a mean of 5.7 years between covariate assessment and actigraphy collection and a mean of 5.9 years of follow-up starting one year after actigraphy. **b** Flow diagram of participants. For each phenotype, subjects were excluded from analysis if they had prior diagnosis of the PheCODE or of any of the PheCODEs defined as exclusion criteria by the PheCODE map. Diagnoses within one year of actigraphy were also excluded, due to the likelihood of disease onset occurring before diagnosis. Source data are provided as a Source Data file.

**Fig. 2:**
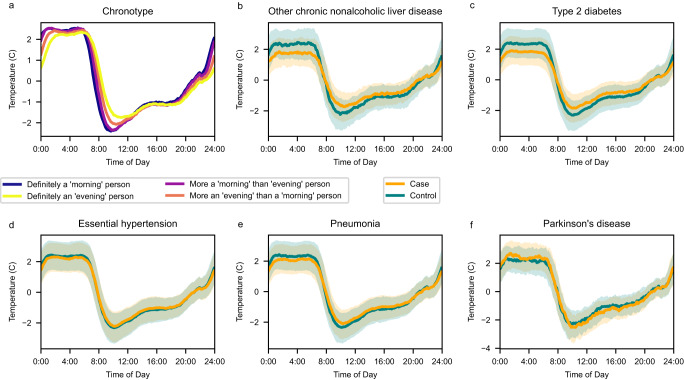
Wrist temperature traces. Wrist temperature traces by (**a**) chronotype (morningness/eveningness), and by case-status in pairs matched by age and sex for (**b**) NAFLD, (**c**) type 2 diabetes, (**d**) hypertension, (**e**) pneumonia, and (**f**) Parkinson’s disease. Please note that the temperature curve for Parkinson’s disease in Panel (**f**) separates from the controls but with opposite directionality compared to the disease conditions displayed in panels (b–e). In plots with just two groups (**b**–**f**), the interquartile range (25th to 75th percentiles) of the population are displayed in shaded regions (controls in blue, cases in yellow and overlap in grayish green). Wrist temperature is normalized so that each individual’s daily median is 0. Source data are provided as a Source Data file.

The UKBB collected 7 days of actigraphy from 103,688 participants (Fig. 1)^20^, with 91,462 participants passing quality control and having all covariates. The wrist actigraph device housed a temperature sensor near the skin. Though its primary purpose was related to accelerometer calibration^17^, we found that the peripheral skin temperature signal (Supplementary Fig. 1) was strong enough to produce a phenotype characteristic of a biological origin. This wrist temperature signal was clearly discernable despite it being modulated (masked) by sleep, ambient temperature conditions, food intake and other confounders. The shape of the UKBB wrist temperature curves matched previously reported curves for peripheral skin measured at the wrist^11^. In the wild, wrist temperature readings characteristically increase with sleep onset, plateau during sleep and then drop suddenly upon awakening—followed by a second smaller peak in the afternoon^11^. The shape depicted in Fig. 2a is consistent with this phenotype. The plateau of maximum wrist temperatures occurs during night hours (Fig. 2a, Supplementary Fig. 2). This shape has been described as running phase-advanced or inverse (anti-phase) to the core body temperature^21^ and to activity (Supplementary Fig. 3). The relationship of cooling down the body core temperature by peripheral vasodilation resulting in heat loss has been proposed as a thermoregulatory mechanism to induce sleep^22^. The peak-to-trough variation in the observed wrist temperature in Fig. 2a–f matches the 6 °C differential between maximum (36.1 ± 0.5 °C) and minimum (30.4 ± 1.7 °C) diurnal temperature fluctuations reported for a cohort of 103 healthy volunteers^23^. The similarity in these data underscores the confidence in the methodology. Roughly 3.5 °C of these peak-to-through oscillations are driven by the circadian clock^21^, the remainder of about 1.5–2 °C is likely driven by behavioral and environmental components. The wrist temperature curves are right shifted for the evening chronotype compared to participants reporting a morning preference (Fig. 2a). This corresponds well with the 2–3 h delay in circadian phase observed between morning and evening chronotypes^24^. This also corresponds to the delay in sleep timing. To underscore the value of wrist temperature as a potential biomarker, we refer to a study in 13 healthy volunteers showing that phase determined by wrist temperature readings correlated strongly (*R* = 0.756) with the circadian phase assessed by dim light melatonin onset (DLMO), which is the gold standard in the field^13^.

### Biorhythms in wrist temperature associate with future diagnoses

**Fig. 3:**
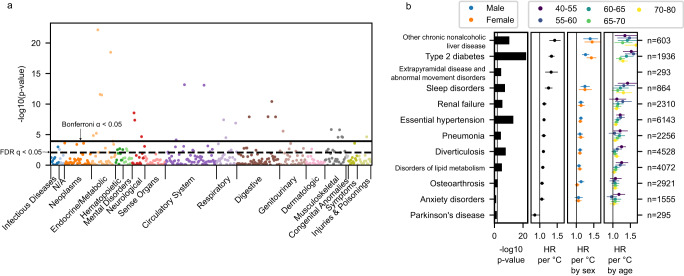
Diurnal Rhythmicity Associate with Diagnoses. To test whether diurnal rhythmicity associate with future disease, a Cox proportional hazards model was performed for each phenotype (PheCODE), using the two-sided Wald test for effects from the wrist temperature amplitude. Individuals with diagnoses prior to the actigraphy measurement were excluded as well as those with a new diagnosis code within the first year following actigraphy. **a** Manhattan plot-style display of phenome-wide results. In many phenotypes, weaker wrist temperature rhythms are associated with future onset of disease. Uncorrected *p*-values are plotted along with the solid line showing Bonferroni-correction significance threshold for *α* ≤ 0.05 and the dashed line showing the Benjamini-Hochberg false discovery rate (FDR) at 0.05 cutoff. Phenotypes range from *n* = 200 events (diagnoses) to *n* = 6728 events (exact *n* values in Supplementary Data 1). **b** A selection of significant phenotypes shown in detail. Left: significance of the overall effect size (irrespective of age and sex). Right: three panels, effect sizes of the overall model, by sex model, and by-age model. Effect sizes were measured as the hazard ratio (HR) per 1 °C decrease in the wrist temperature amplitude. Circles denote the point estimate of HR and lines denote their 95% CI. Number of events (first diagnoses) *n* for each phenotype are shown on the far right. Extrapyramidal disease and Parkinson’s disease had insufficient cases to run by age and sex. Anxiety disorders and Parkinson’s disease are slightly above the *q* < 0.05 threshold, at *q* = 0.20 and 0.11, respectively. No phenotypes displayed significant differences by sex or by age after correction for multiple testing (*q* ≥ 0.48 for all phenotypes). Source data are provided as a Source Data file.

Disease phenotypes were extracted from medical records and grouped according to the PheCODE Map^25,26^. To investigate how well wrist temperature amplitude associates with future diagnoses, Cox proportional hazards models were computed with each PheCODE diagnosis as an endpoint and the temperature amplitude as the independent variable. To exclude participants with subclinical disease at the time of actigraphy, we limited enrollment to those participants whose first recorded diagnostic event was at least one year after their actigraphy assay. Of the 425 PheCODEs with at least 200 cases, a total of 73 (17%) reached significance at a Benjamini-Hochberg false discovery rate (FDR) of *q* < 0.05 (Fig. 3a, Supplementary Data 1). In each of eight categories (neoplasms, neurological, digestive, genitourinary, respiratory, musculoskeletal, circulatory system, and endocrine/metabolic conditions) five or more PheCODEs were significant at this level, highlighting the importance of interrogating across the human phenome. The more conservative Bonferroni family-wise error rate (FWER) of α < 0.05, (*p* < 0.00012, Fig. 3a), identified a total of 26 (6.1%) PheCODEs. Here, the most significant associations represent common chronic disease phenotypes that have been associated with circadian disruption^6,27^.

The following vignettes explore selected disease associations in more detail. A two-standard deviation (SD) decrease (1.8 °C) in wrist temperature amplitude (HR = 1.91 [1.58–2.31, 95% CI], *q* = 2.3 × 10^−9^, *n* = 603 events) is associated with nearly double the rate of nonalcoholic fatty liver disease (NAFLD) disease. The most common billing codes for this PheCODE were steatosis (*n* = 533 for ICD10 K76.0) and cirrhosis (*n* = 79 for ICD10 K74.6). This corroborates the known loss in diurnal variance of distal skin temperature in cohorts of patients with liver cirrhosis compared to controls^28^. Consequently, the distal-proximal temperature gradient indicative of heat dissipation is blunted, which contributes to the highly prevalent sleep disruptions reported for cirrhotic patients^29^, among other proposed mechanisms such as displaced melatonin secretion^30^. This raises the question of whether repeated weeklong wrist temperature readings, assessing diurnal variability in the thermoregulatory system, constitutes a valid digital biomarker to monitor disease progression.

Onset of diabetes has long been associated with circadian clock disruption^31^. The ensuing peripheral vascular disease and neuropathy impair thermoregulation specifically at night^32,33^. The resulting reduction in the distal temperature amplitude has been observed in diabetic patients before polyneuropathy manifested^32^, suggesting that altered peripheral temperature rhythms identify patients early at risk of disease progression. In the UKBB data, wrist temperature rhythms have an HR for type 2 diabetes mellitus of 1.69 (1.53–1.88, 95% CI, *q* = 2.9 × 10^−20^, *n* = 1,936 events).

Wrist temperature rhythms also had large associations with risk of hypertension (HR = 1.23 [1.17–1.30], *q* = 8.4 × 10^−12^, *n* = 6143) and in disorders of lipid metabolism (HR = 1.16 [1.09–1.24] *q* = 1.4 × 10^−4^, *n* = 4072), consistent with the link to metabolic syndrome^34^.

A 2 SD lower wrist temperature amplitude indicated a 67% higher risk of extrapyramidal disease and abnormal movement disorders (HR = 1.67 [1.32–2.11], *q* = 4.4 × 10^−4^, *n* = 293). Diurnal variation in dopamine neurotransmission is well known^35^ with evidence for functional consequences. Drug-induced evocation of extrapyramidal symptoms, akathisia and dystonia, in patients with schizophrenia was more severe at night compared to the morning hours^36^. This relationship is likely modulated by the high degree of inter-individual variability in dopamine D2 receptor density^37^, which, with its role in central thermoregulation, may lead to the high association of wrist temperature for susceptibility to extrapyramidal disease.

Psychiatric disorders are commonly associated with altered circadian rhythms^38^. In the present study, anxiety disorder was one of the few mental PheCODEs with more than 200 cases to show a trend (HR = 1.11 [0.99–1.24], *q* = 0.20, *n* = 1555) (Fig. 3b, Table 1).

**Table 1:** Hazard ratios for select diagnoses

PheCODE	HR at 2 SD	HR at 1 °C	*N*
NAFLD (Other chronic nonalcoholic liver disease)	1.91 (1.58–2.31)	1.43 (1.28–1.58)	603
Type 2 diabetes	1.69 (1.53–1.88)	1.34 (1.26–1.42)	1936
Extrapyramidal disease and abnormal movement disorders	1.67 (1.32–2.11)	1.33 (1.16–1.51)	293
Sleep disorders	1.50 (1.3–1.74)	1.25 (1.15–1.36)	864
Renal failure	1.25 (1.14–1.37)	1.13 (1.07–1.19)	2310
Essential hypertension	1.23 (1.17–1.3)	1.12 (1.09–1.16)	6143
Diverticulosis	1.20 (1.13–1.28)	1.11 (1.07–1.14)	4528
Pneumonia	1.22 (1.11–1.33)	1.11 (1.06–1.17)	2256
Disorders of lipid^a^ metabolism	1.16 (1.09–1.24)	1.09 (1.05–1.13)	4072
Osteoarthrosis	1.12 (1.04–1.21)	1.06 (1.02–1.11)	2921
Anxiety disorders	1.11 (0.99–1.24)	1.06 (1.0–1.12)	1555
Parkinson’s disease	0.76 (0.59–0.97)	0.86 (0.75–0.98)	295

From the Cox proportional hazards models, wrist temperature amplitudes associated with disease outcomes. Twelve of the largest significant effect sizes are shown, as hazard ratios comparing mean to two standard deviations (SD) below, corresponding to 1.8 °C, below mean amplitude, or comparing to 1 °C below the mean, along with the number of events (cases). See also Supplementary Data 1 for full results.

^a^PheCODE Map^25, 26^ uses the term “lipoid”.

Associations with the opposite directionality occurred in just a few PheCODEs. Decreased wrist temperature rhythms were associated with decreased future diagnoses for Parkinson’s disease (HR = 0.76 [0.59–0.97], *q* = 0.11, *n* = 295) (Fig. 2f), and with Raynaud’s syndrome (HR = 0.63 [0.51–0.79], *q* = 0.001, *n* = 295). These are unexpected findings. However, both of these diseases are marked by impaired thermoregulation^39,40^, linked in Parkinson’s to alpha-synuclein pathology in the CNS^40^ and in Raynaud’s to sympathetic nervous system and α2C adrenoreceptor dysregulation in the small blood vessels of the digits^41^. In Parkinson’s, the peripheral temperature trace is likely altered by episodic hyperhidrosis^39^ particularly in the dysautonomic subtype^42^. The accelerometry trace in Parkinson’s patients indicated lower diurnal variation and physical activity (Supplementary Fig. 3), an observation associated with elevated risk of disease^43^. Here, time-specific deep phenotyping studies, like those performed by the human chronobiome project^44^, are necessary to disentangle disease mechanisms.

### Sex- and age-dependent effects in wrist temperature rhythms

Trends for sex-dependent differences emerged for several disease phenotypes. Hernias, degenerative joint diseases, reflux esophagitis and colorectal cancer showed a weak interaction between sex and wrist temperature amplitude (*p* < 0.05); however, to the extent that these are real, there was insufficient power to overcome correction for multiple testing (*q* > 0.5 for all PheCODEs).

Similarly, trends (*p* < 0.05) for age-dependent differences were present in age-related diseases, such as diabetes, cognitive disorders, cataract, neoplasms, heart valve disease and diverticulosis (*q* > 0.48 for all PheCODEs).

### Wrist temperature rhythms and all-cause mortality

Overall, subjects with lower amplitude had increased risk of all-cause mortality (*p* = 0.002, *n* = 3061 deaths HR = 1.14 [1.04–1.23, 95% CI] for a 2 SD decrease in amplitude). Age, fitted as a linear relationship with the outcome, was controlled in that model. To confirm age-independence of this relationship, the association between decreased amplitude and mortality remained significant when age categories of under and over 65 years of age were analyzed separately (*p* = 0.037, HR = 1.10, *n* = 759 deaths for <65 years of age compared to *p* = 0.018, HR = 1.06, *n* = 2302 deaths for >65 years of age). Interestingly, mortality associations were not consistently significant among sub cohorts (see Methods), possibly driven by lower death counts or covariates such as recruitment center.

### Temperature biorhythm atlas

**Fig. 4:**
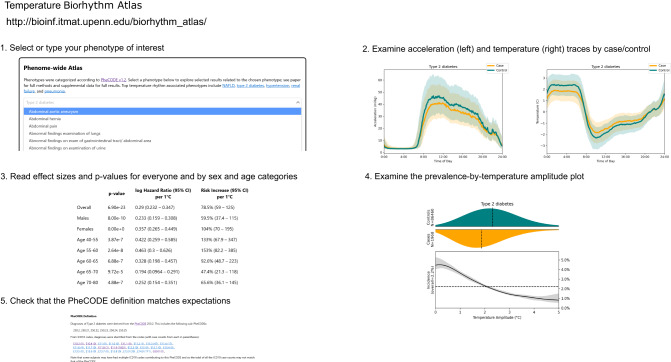
Temperature biorhythm atlas guide. We have made the results of this study easily explorable on a web-based atlas (http://bioinf.itmat.upenn.edu/biorhythm_atlas/). Users select a phenotype (PheCODE) of interest from a drop-down list. Then, they are presented with results pertaining to that phenotype. First, average traces by time-of-day are shown for matched case/control pairs, as in Fig. 2. Next, effect sizes and statistical significance in a tabular format, broken down by sex and age (for phenotypes with sufficient case counts in each category). Then a plot of distributions of wrist temperature amplitudes in both the cases and controls, along with a plot of the risk of the disease stratified by temperature amplitude rhythms. Non-constant risk rates indicate an association of rhythm with disease. Lastly, details about the definition of cases and controls for the phenotype are given, including the specific ICD-10 codes (with case counts) used to identify cases, as well as the exclusion criterion. These allow investigators interested in a single phenotype to quickly assess it for connections to diurnal rhythm disruption.

To enable phenome-wide access to the results, we created a comprehensive temperature biorhythm atlas to visualize and quantify how the diurnal phenotypes of wrist temperature modulate the rate of diagnosis for a future disease of interest following the International Classification of Diseases 10th Revision (ICD-10) codes. Diurnal acceleration traces are provided as visual reference. This atlas (http://bioinf.itmat.upenn.edu/biorhythm_atlas/) serves as a resource for clinicians, researchers, and the public to consider temperature biorhythms as a potential digital biomarker for the future onset of diseases (Fig. 4).

## Discussion

We identified 73 out of 425 (17%) disease phenotypes across the human phenome that were associated with decreased wrist temperature amplitudes, 26 of which (6.1%) were highly significant under more stringent criteria. By introducing a latency period of one year, we eliminated potentially undiagnosed mild or subclinical disease conditions present during the monitoring period which might have had a dampening effect on the temperature rhythms. Among the highly associated diseases were NAFLD, diabetes mellitus, hypertension, asthma, chronic airway obstruction, lipid disorders, chronic liver disease, renal failure, and pneumonia. These include many of the chronic conditions responsible for 90% of the $4.1 trillion spent annually on health care in the US^45^.

The strength of our phenome-wide approach is the systematic, disease-specific quantification of disrupted wrist temperature rhythms which so far has relied on annotating disease phenotypes separately by domain experts^46^. These results invite us to think about how interventional studies could characterize the relationship between low amplitude wrist temperature rhythms and associated future onset of diseases. As this relationship is driven by circadian, sleep-wake behavior and environmental components, interventions may target different aspects. This is important when testing the sensitivity and specificity of diurnal skin temperature amplitude as a disease biomarker. Clinical studies could, for example, deploy high heat capacity mattresses^47,48^ as an intervention to test whether this strategy could strengthen robustness of temperature biorhythms, and to discern underlying changes in the molecular fingerprint. Novel wearable omics devices that confer low patient burden can complement this to generate mechanistic insight^49^. These are potential steps to disentangle causal relationships where circadian disruption raises disease susceptibility and severity while many diseases disrupt circadian rhythms^27^. These insights are expected to empower a more nuanced personalization of circadian health^50^, balancing lifestyle risk factors and disease pathology.

Thermoregulation in humans enables body temperature to stay within a narrow, tightly controlled range where physiological processes run most efficiently. Core and peripheral body temperature are inversely coupled to regulate body temperature by balancing heat production and loss^10,11^. Deviation from this is controlled during a febrile response to fight pathogens, or uncontrolled in cases of prolonged extrinsically caused hyper- or hypothermia, often resulting in multi-organ failure^51,52^. The circadian aspect of thermoregulation is achieved through diurnal oscillations of the temperature set point in the hypothalamic preoptic area and controlled by the ‘master’ clock in the suprachiasmatic nucleus (SCN)^53^. Thermogenic and heat-dissipative processes modulate the set point throughout the course of 24 hours typically resulting in peak core temperatures in the afternoon with the nadir reached at the end of the sleeping phase^11^. These oscillatory temperature cues are likely picked up in the periphery to entrain, for example, sets of hepatic gene transcripts independent of peripheral liver clocks^54^. This was suggested in a mouse model, engineered to differentiate between central and peripheral entrainment cues, where the authors showed that about 10% of rhythmically expressed liver genes were not under control of hepatocyte clocks but instead relied on oscillatory systemic signals^54^.

Of course, other mechanisms are at play. Temperature-dependent polyadenylation through RBM3, a cold-inducible RNA-binding protein, has been suggested to contribute to cell reprogramming during a stress response^55,56^. An explanation for some of the strong associations observed in this study might be that small amplitude temperature rhythms diminish thermosensitive gene regulation, which in combination with disease-specific perturbations, like those observed for diabetic neuropathy,^32,33^ lead to the emergence of specific disease phenotypes. We suggest that comprehensive studies, such as the one piloted in the human chronobiome project^44^, are necessary to untangle mechanistic relationships. Time-integrated transomic assessments seem to be necessary to tease out how thermoregulatory rhythms entrained, for example, by microbiota^57^ fit into the picture.

We found a strong association between dampened temperature rhythms and mortality. The mortality rate was increased by 14% in those who displayed a wrist temperature amplitude that was diminished by two standard deviations (1.8 °C); and the literature supports this association. For example, lifespan was increased in transgenic mice by 12% in males and 20% in females with strengthened temperature amplitudes^58^. And overexpression of uncoupling protein 2 in hypocretin neurons (Hcrt-UCP2) of these animals reduced core body temperature by 0.3–0.5 °C accompanied (though this was not measured) by a presumable increase in peripheral temperature through the coupling of core and periphery in the thermoregulatory sleep model^10^.

One limitation of this study is that the wrist temperature readings were collected from a sensor enclosed within the wrist-worn actigraphy device where the sensor is separated from direct skin contact by a few millimeters of plastic. Our results indicate that the temperature traces in the UKBB study capture biological signal compellingly and agree with field studies^13,23,32^. Further support comes from a study where the skin temperature measurements did not correlate with ambient temperature readings from a second device placed nearby on the participants’ external clothing, suggesting that a device worn on the wrist sufficiently captures the biological signal and is little contaminated by ambient temperature^32^. Nevertheless, ambient temperature fluctuations should be considered as a potential factor. Here, wearables with discrete temperature tracing^59^ offer complementary monitoring of thermoregulation in future efforts. A second limitation is that disease phenotypes are collected from in-patient hospital diagnostic codes. Therefore, some phenotypes may be recorded significantly later than actual disease onset and others may be missed from the dataset entirely. This was partially corrected for by excluding subjects according to prior diagnoses of any related disease, derived from including self-reported medical conditions at the initial assessment. Another limitation is that temperature rhythms are confounded by BMI as illustrated in Supplementary Fig. 2. Although we included BMI as a covariate in the models, its measurement preceded actigraphy by about five years, so it served as an incomplete control. Menstrual phases modulate peripheral temperature readings^60^, but the lack of contraceptive or menstrual cycle phase data precluded any further analyses. Data on menopause status was collected but not at the time of accelerometry for most UKBB participants. In a small set of subjects recalled for imaging at around the same time as the actigraphy was taken, about 95% of females were post-menopausal. Lastly, since cosine curves do not capture the exact shape of diurnal wrist temperature variation, future approaches could include using higher harmonic terms^61^ or highly flexible approaches such as functional principal component analysis^62^.

In conclusion, we established that decreased wrist temperature amplitudes associate with disease risk in participants of the UKBB, suggesting peripheral thermoregulation as a digital biomarker.

## Methods

Data for this study was obtained from the UK Biobank Resource under Application Number 50398 (approval date June 18th, 2019).

### Actigraphy in the UK Biobank

The UKBB collected 7 days of actigraphy from 103,688 participants (Fig. 1)^20^ roughly from June 2013 to December 2015 using the Aximetry AX3 wrist-worn device on dominant wrists. The data were calibrated and processed using an existing pipeline^20,63–65^ modified slightly to better accommodate daylight savings time changes and to output light and temperature readings from an on-device thermometer. A total of 92,325 participants passed quality control after exclusions based on failed calibration (*n* = 2826), insufficient data (*n* = 4258) or crossing over daylight savings time (*n* = 4279), following prior experiments^7,8,20^. A further *n* = 873 subjects were excluded for missing data from the covariates for a total of 91,462 with no missing data who were thus selected for analysis. The overall correlation between physical activity and wrist temperature was weak (Spearman *r* = 0.21), indicating that temperature is a distinct measure despite being recorded from the same device as motion.

In addition, repeated measurements across seasons were collected for 3197 participants. This data is available but has not yet been reported on in detail. We leveraged these repeated datasets to account for seasonality across participants and to determine the variance of temperature amplitudes.

### Wrist temperature

To convert temperature into degrees Celsius, we corrected the values reported by our actigraphy pipeline as *T* = (500*X* − 2550)/256, where *X* is the original value reported in arbitrary units and *T* is the temperature in Celsius. This makes our reported values match those used in a prior, thorough investigation of these temperature values^17^ which corrected a flaw in earlier processing pipelines.

Non-wear time was determined via the acceleration signal as part of the existing processing pipeline^20^. All temperature readings during detected non-wear time were treated as non-physiological and regarded as missing values.

Diurnal rhythms were computed via cosinor fits^66^ of the wrist temperatures (Supplementary Fig. 1) in each participant, and amplitude parameter were extracted from each fit. These amplitudes were measured in degrees Celsius and give the difference between mesor (mid-line) and peak values of the fit. Peak-to-trough values reflect twice the amplitude. Cosinor fits have been validated for use in measuring rhythms in both distal skin temperature and core body temperature^14,67^.

Since temperature is expected to be highly seasonally variable, we corrected for season via a cosinor fit over time-of-year on the log temperature amplitude.

Anyone with a reported temperature amplitude above 10 degrees Celsius was dropped as being non-physiological. Only 2 individuals (out of 92,322) exceeded this threshold and were dropped from further analysis. Furthermore, only 28 subjects exceed a more stringent threshold of 6 degrees Celsius, indicating that non-physiological effects are likely not a large influence.

Since each actigraphy device was used by multiple individuals, we examined the device-effect to check for calibration problems. We identified three “clusters” of device ids with distinctly different values reported, Supplementary Fig. 4 top. The clusters were separated according to device IDs below 7500, between 7500 and 12,500, between 12,500 and 20,000, or above 20,000. The highest device ID cluster occurred only during repeat measurements data, used in this study only to correct for seasonality. The temperature amplitude was corrected by a multiplicative scaling of each cluster such that the median of each cluster was set to be equal to the overall median. We observed that these clusters were associated with season of measurement but not with other covariates such as sex, BMI, birth year, or Townsend deprivation index.

After this correction, we assessed calibration quality, by comparing the mean values among all individuals who used the same device to the means of a random permutation of the device ids. We expect measures without calibration problems to have a comparable device-level distribution to that generated by the random permutation of device IDs. The mean temperature shows strong device-specific bias, Supplementary Fig. 4c, indicating a lack of temperature-sensor calibration. However, the cosinor amplitude of temperature measures are robust and show little device-specific bias, Supplementary Fig. 4c.

### Phenotypes

Diagnosis events were assessed in subjects starting one year after actigraphy, for a mean follow-up time of 5.9 years (4.8–7.3 min-max), see Figure1a. Events were identified from inpatient hospital record ICD-10 codes, which were grouped into phenotypes (PheCODEs)^25,26^ using the PheCODE Map v1.2b1. The PheCODE Map provides, for each phenotype, exclusion criteria denoting similar conditions that may indicate likelihood of undiagnosed patients of the phenotype under consideration. For example, for the renal failure analysis, subjects were excluded if they had a history of acute glomerulonephritis; renal sclerosis (unspecified); disorders resulting from impaired renal function; small kidney of unknown cause; or infections of kidney, as well as renal failure itself. These exclusions are determined by in-patient hospital diagnosis codes (mapped to PheCODEs by the PheCODE Map v 1.2b1 and v1.2 for both ICD-10 and ICD-9 codes) and for self-reported medical conditions that were determined at initial assessment (mapped to PheCODEs by expert opinion, C.S.). All subjects meeting any of the exclusion diagnoses for a phenotype at the end of the one-year lag period (i.e., one year after actigraphy) were excluded from the analysis of that phenotype as likely or potential prior cases. Full details of exclusions are reported for each phenotype in Supplementary Data 1 as well as on the online atlas. Subjects excluded from analysis of one PheCODE are not excluded from others, unless meeting the exclusion criteria of those PheCODEs as well.

A total of 451,994 distinct patient diagnoses (mean 4.9 per participant) and 3,061 deaths (3.3%) were recorded among these participants at the time of data download on February 23, 2022 (Fig. 1a).

### Proportional hazards models

A Cox proportional hazards model was performed for each PheCODE with ≥200 cases, based on a power analysis performed via simulation to establish a 0.2 log (hazard ratio) effect size at 80% power. The endpoint was diagnosis with the PheCODE, censored by death or end of data collection. The timescale utilized was years since actigraphy measurement. The independent variable was temperature amplitude. The model included covariates for sex (male/female, reflecting NHS records at recruitment or subsequent participant-reported updates), ethnicity (white/other, since 97% are white^8^), smoking history (Prefer not to answer/Never/Previous/Current)), age at the time of actigraphy measure (40-55/55-60/60-65/65-70/70-80), BMI at assessment (continuous), college education (yes/no), Townsend deprivation index (continuous), alcohol consumption (Prefer not to answer/Daily or almost daily/Three or four times a week/Once or twice a week/One to three times a month/Special occasions only/Never)) and self-reported overall health (Do not know/Prefer not to answer/Excellent/Good/Fair/Poor), determined at initial assessment. Survival analysis was performed with the same model but with death as endpoint. A complete-case analysis was performed on 91,462 subjects with complete data (Fig. 1b). All covariates were assessed at initial enrollment in the UKBB, which had a mean of 5.7 (2.8–8.7 min-max) years before actigraphy collection. Results were computed in terms of the hazard ratio (HR) and its robust standard errors, performed by the R package *survival*^68^. In text, we report HR per 2 SD of amplitude, corresponding to 1.8 °C; 16% of individuals have 1 SD below mean amplitude and 1.1% of the population have 2 SD below mean amplitude.

To check for sex-specific and age-dependent effects, further models were generated including interaction terms for sex and age at the time of actigraphy measurement. All *p-*values were computed by two-sided Wald tests.

### Appropriateness of the proportional hazard model assumptions

To assess the appropriateness of the model assumptions, the top 12 phenotypes were examined in detail. For these, the Schoenfeld residuals were plotted and were tested for non-zero slope by the cox.zph function. For variables displaying significant (*p* < 0.01) non-constant violations, a second model was rerun including a linear time-interaction term (if quantitative) or a stratification term (if categorical) which was then compared to the original model to assess impact. To validate the linearity of the effect, a third model was run which included a penalized smoothing spline over the amplitude with 3 degrees of freedom. Effects were then plotted and inspected for non-linearity. Lastly, models were rerun while treating death as a competing outcome. All results were confirmed to show only small changes in hazard ratios of the wrist temperature amplitude compared to the original model (see Supplementary Data 2).

### Validation and multiple hypothesis testing

We further validated that the results were consistent within two sub cohorts. These cohorts were chosen based off the actigraphy device ID clusters (see Wrist Temperature section) with cluster A as one cohort (*n* = 53,912 complete cases) and the two smaller clusters, B and C, were combined as a second cohort (*n* = 28,500 complete cases). The expectation was that actigraphy device cohort may correlate with unknown confounders such as the study center responsible for handling them and therefore represent good choices to verify robustness of the analysis.

All results presented in this paper were consistent between all three cohorts in phenotypes with large sample sizes. For example, essential hypertension has HR of 1.23 (1.17–1.3 95% CI) in the full cohort, 1.22 (1.14–1.30) in sub cohort A and 1.26 (1.15–1.38) in sub-cohort B + C. Likewise, type 2 diabetes has HRs of 1.69 (1.53–1.88), 1.65 (1.45–1.87), and 1.79 (1.49–2.14). The results tables from this manuscript were also generated for the other cohorts and are provided in Supplementary Data 3 for comparison.

Notably, all-cause mortality had *p* = 0.4 in the cohort B + C, though it showed consistent effect direction (HR = 0.031 per degree C decrease, *n* = 911 deaths) with the full cohort and cohort A.

To control for the large number of statistical tests performed, *p*-values have been adjusted where appropriate as Benjamini-Hochberg *q*-values to control the false discovery rate.

### Reporting summary

Further information on research design is available in the Nature Portfolio Reporting Summary linked to this article.

### Supplementary information

Supplementary Information File

Description of Additional Supplementary Files

Supplementary Data 1

Supplementary Data 2

Supplementary Data 3 Zip

Reporting Summary

### Source data

Source Data

## Data Availability

Data for this study was obtained from the UK Biobank Resource under Application Number 50398. The data is available to bona fide researchers for a fee from the UK Biobank. Since this data is not widely available, a small, simulated dataset that mimics the characteristics of the real data is available along with our source code (see Code Availability section) and can be used to demonstrate or test our analyses. Source data are provided with this paper.

## Code availability

The source code for all statistical tests, and generated figures used in this analysis is available at GitHub^69^ and includes simulated data to test the statistics and analyses, with instructions in the README.md file. The modified pipeline for processing the raw actigraphy data is available at GitHub^70^.
