# Supplementary material for: Diurnal rhythms of wrist temperature are associated with future disease risk in the UK Biobank
Source: Nat Commun. 2023 Aug 24;14:5172. doi: 10.1038/s41467-023-40977-5 (PMC10449859; doi:10.1038/s41467-023-40977-5)
Supplement: Supplementary file 1 — Supplementary Information File [file 41467_2023_40977_MOESM1_ESM.pdf]

# Supplementary Information

**Title**

## Diurnal rhythms of wrist temperature are associated with future disease risk in the UK Biobank

**Author List**

**Thomas G. Brooks[1*], Nicholas F. Lahens[1], Gregory R. Grant[1,2], Yvette I. Sheline[3,4,5], Garret A. FitzGerald[1,6,7], & Carsten Skarke[1,6*]**

**Affiliations**

[1]Institute for Translational Medicine and Therapeutics (ITMAT), University of Pennsylvania Perelman School of Medicine, Philadelphia, PA, USA

[2]Department of Genetics, University of Pennsylvania Perelman School of Medicine, Philadelphia, PA, USA

[3]Department of Radiology, University of Pennsylvania Perelman School of Medicine, Philadelphia, PA, USA

[4]Department of Psychiatry, University of Pennsylvania Perelman School of Medicine, Philadelphia, PA, USA

[5]Department of Neurology, University of Pennsylvania Perelman School of Medicine, Philadelphia, PA, USA

[6]Department of Medicine, University of Pennsylvania Perelman School of Medicine, Philadelphia, PA, USA

[7]Department of Systems Pharmacology and Translational Therapeutics, University of Pennsylvania Perelman School of Medicine, Philadelphia, PA, USA

*Corresponding Authors:

Thomas G. Brooks, PhD (thobr@sas.upenn.edu) & Carsten Skarke, MD

(cskarke@pennmedicine.upenn.edu) Institute for Translational Medicine and

Therapeutics (ITMAT), University of Pennsylvania Perelman School of Medicine,
Smilow Center for Translational Research 10-101, 3400 Civic Center Blvd, Philadelphia,
Pennsylvania 19104, USA

**Supplementary Results**

Participants

Other studies have found a “healthy volunteer” effect in the UKBB where participants have greater health than the overall population [6]. This bias increased among participants enrolled in actigraphy collection, who tended to have overall fewer ICD10 codes in their medical records (mean±SD 9.3±11.5 per person) than did those who did not enroll in actigraphy (11.7±14 per person), counting all diagnoses, not just those used for the prospective study. Compared to the UKBB overall, individuals with actigraphy tended to be slightly more likely to be Caucasian, female, younger, and with lower BMI (Table S 2).

Since much of the UK Biobank population are retirees, we restricted it to those age 65 or less and computed the temperature cosinor fit in just weekdays or just weekend days. The temperature amplitude was higher on weekends (0.3°C difference, p = 0 by Wilcoxon signed-rank test, n=49,707). One limitation of this is that with just a single week of data, cosinor fits of the weekend days are drawn from a very limited amount of data and may not be representative for the individual.

Chronotype

The time-of-day preference was surveyed through a chronotype questionnaire. Individuals who identified as definitely a morning person had 0.17 °C (0.147-0.193 °C, 95% CI) higher temperature amplitudes than those who identified as definitely an evening person ($p$ = $7 \times 10^{-46}$, OLS regression with the same covariates as the main Cox proportional hazards analysis). However, we did not find an interaction of disease with chronotype. For example, in hypertension, the difference between amplitudes of cases

and controls in evening chronotypes was similar to the difference in morning chronotypes (Figure S 2).

Total Diagnosis Count

To investigate multimorbidity and the disease-free subpopulation, we have counted the number of distinct ICD10 codes per individual, irrespective of time of diagnosis. The population was then stratified by number of diagnoses (0, 1-2, 3-5, 6-10, 11-15, 16-25, 25+), see Figure S 5. There is a trend of higher multimorbidity having lower amplitude, however, performing an OLS regression of temp_amplitude against these categories (and using the same set of covariates as in our main model), we only obtain a significant difference from the reference class of 0 codes for the 16-25 and 26+ groups (the 11-15 category has p=0.053). This may indicate a weakened signal by grouping all phenotypes together compared to the phenotype-specific analyses in our main Cox models.

Stability assessment

Repeat assessments of the 7-day actigraphy were performed on a subset of individuals (*n*=3,197; *n*=2,339 with five total assessments, 727 with four, 93 with three and 38 with two). To assess the stability and repeatability of the measured temperature rhythm, we computed amplitudes on each of these repeats. Among individuals with a measurement in all four seasons, the mean intra-individual standard deviation was 0.70°C for the temperature amplitude (after correcting for seasonality and device clusters, described above), compared to the mean amplitude was 2.3°C. The intraclass correlation (ICC) among individuals with all four seasonal repeats is ICC(3,1)=0.54 (the ICC for a single measurement with seasonal measurements, using the Shrout and Fleiss convention [7].

This increases to ICC(3,k)=0.83 (the ICC of the mean of all four seasonal values). If instead seasonality is not corrected for in the temperature readings, then we have ICC(3,1)=0.53 and ICC(3,k)=0.81. Therefore, correction for seasonality only slightly improves stability of the amplitude measure, and even more helpful would be to use the average of four week-long measurements taken throughout the year instead of a single week-long measurement. Another factor to improve stability could be the use of more precisely calibrated temperature monitors.

## Supplementary Figures

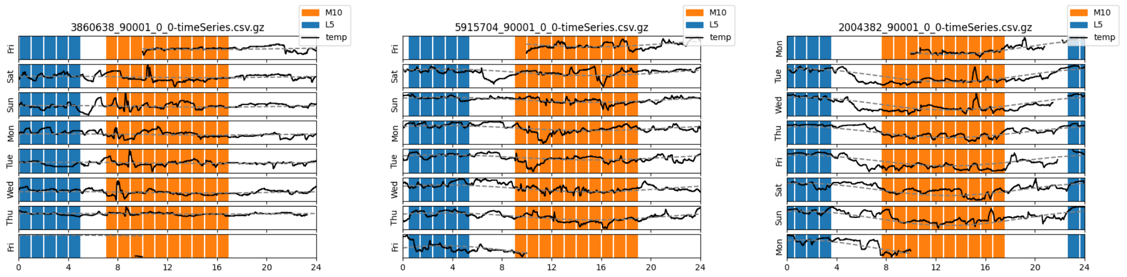


**Supplementary Figure 1 – Example temperature traces**

Example temperature trace from three participants with low (left), typical (middle), and high (right) temperature amplitude. The M10 period (ten hours of highest average activity) and L5 period (5 hours of lowest average activity) highlighted for reference. Cosinor fits for temperature are given in dashed gray line.

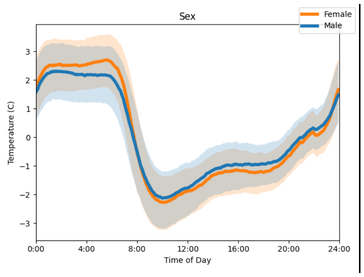


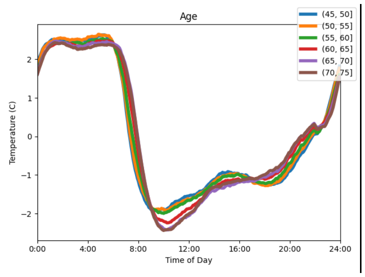


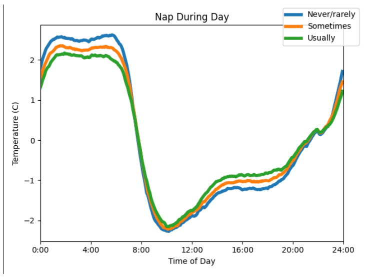


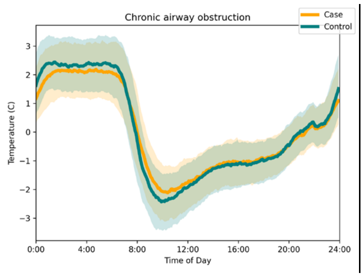


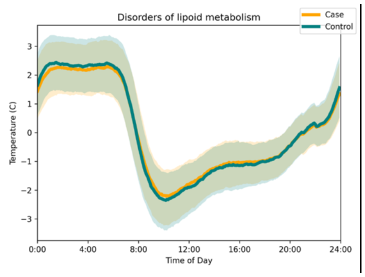


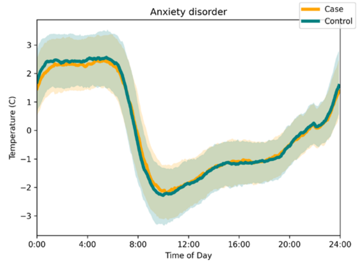


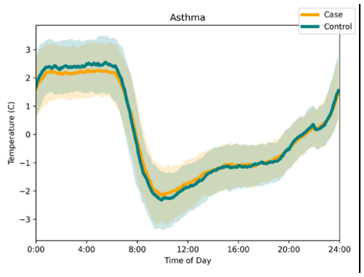


## Morning Person

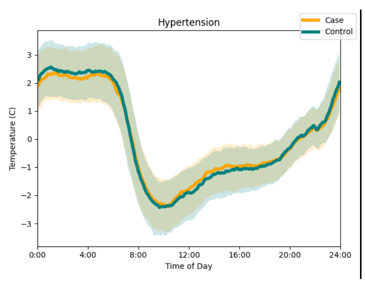


## Evening Person

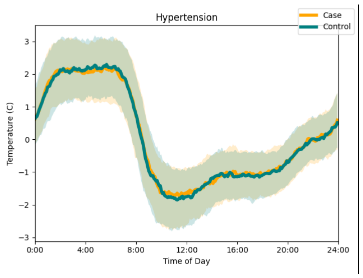


## Morning Person: BMI

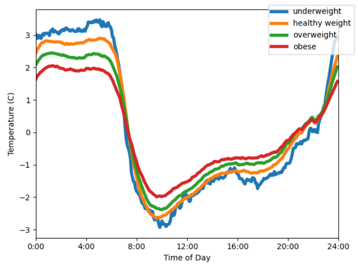


## Evening Person: BMI

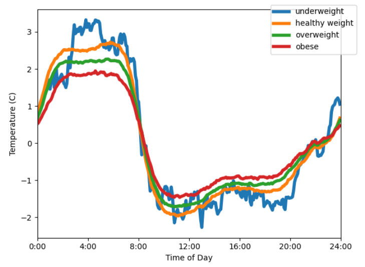

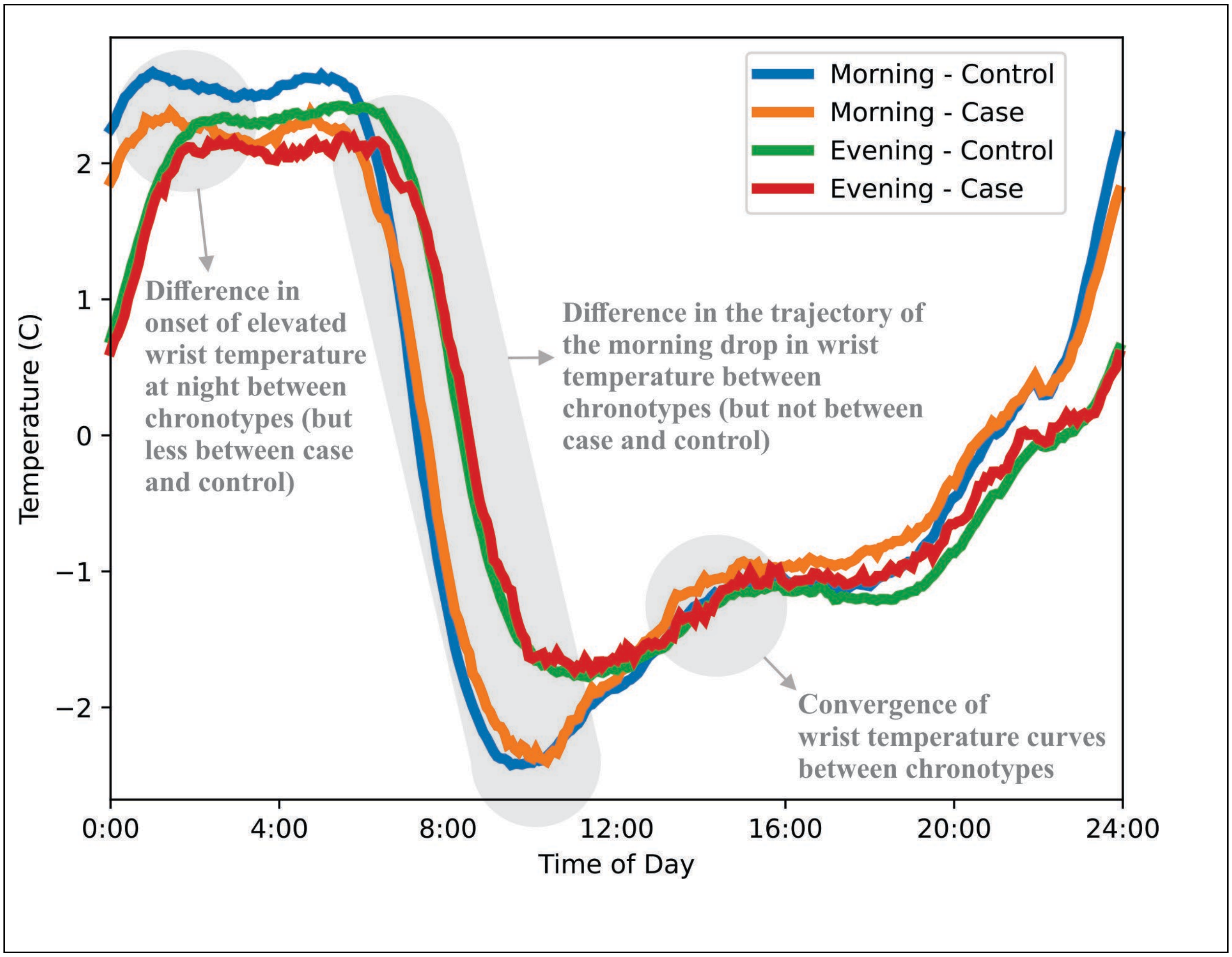


**Supplementary Figure 2 – Detailed wrist temperature traces**

Median wrist temperature traces comparing cases and controls for differences by sex, age, self-reported napping during the day as well as by disease condition for cases with chronic airway obstruction, disorders of lipoid metabolism, anxiety disorder, and asthma. For plots with just two groups, interquartile ranges ($25^{th}$ to $75^{th}$ percentiles) of the population are displayed in shaded regions (controls in blue, cases in yellow and overlap in grayish green). Temperature is plotted relative to an individual's overall median. To

explore associations of ‘chronotype of person’ with ‘rhythm profile of disease’, we plotted the peripheral temperature trace for patients with hypertension and controls of self-reported morning chronotype. Hypertensive and normotensive participants self-reporting an evening chronotype showed a later onset of elevated wrist temperature at night compared to hypertensives and normotensives with a morning type. Similarly, the morning drop in wrist temperature occurred later in evening compared to morning chronotypes. Interestingly, wrist temperatures for morning and evening chronotypes converged between noon and late evening. Notably, wrist temperatures reached consistently lower plateaus at night in hypertensives of both morning and evening type compared to their respective controls. In summary, this suggests an absence of interaction between chronotype and hypertension on wrist temperature curves (**Figure S 2**, last plot). Increase in body weight (BMI categorized as <18.5 (underweight), 18.5-25 (healthy weight), 25-30 (overweight), and >30 (obese)) associates with a loss of amplitude in skin temperature which tracks with self-reported morning and evening chronotype but without indication of an interaction. Note that the underweight category is small and so has high variance. See Figure S3 for corresponding acceleration plots.

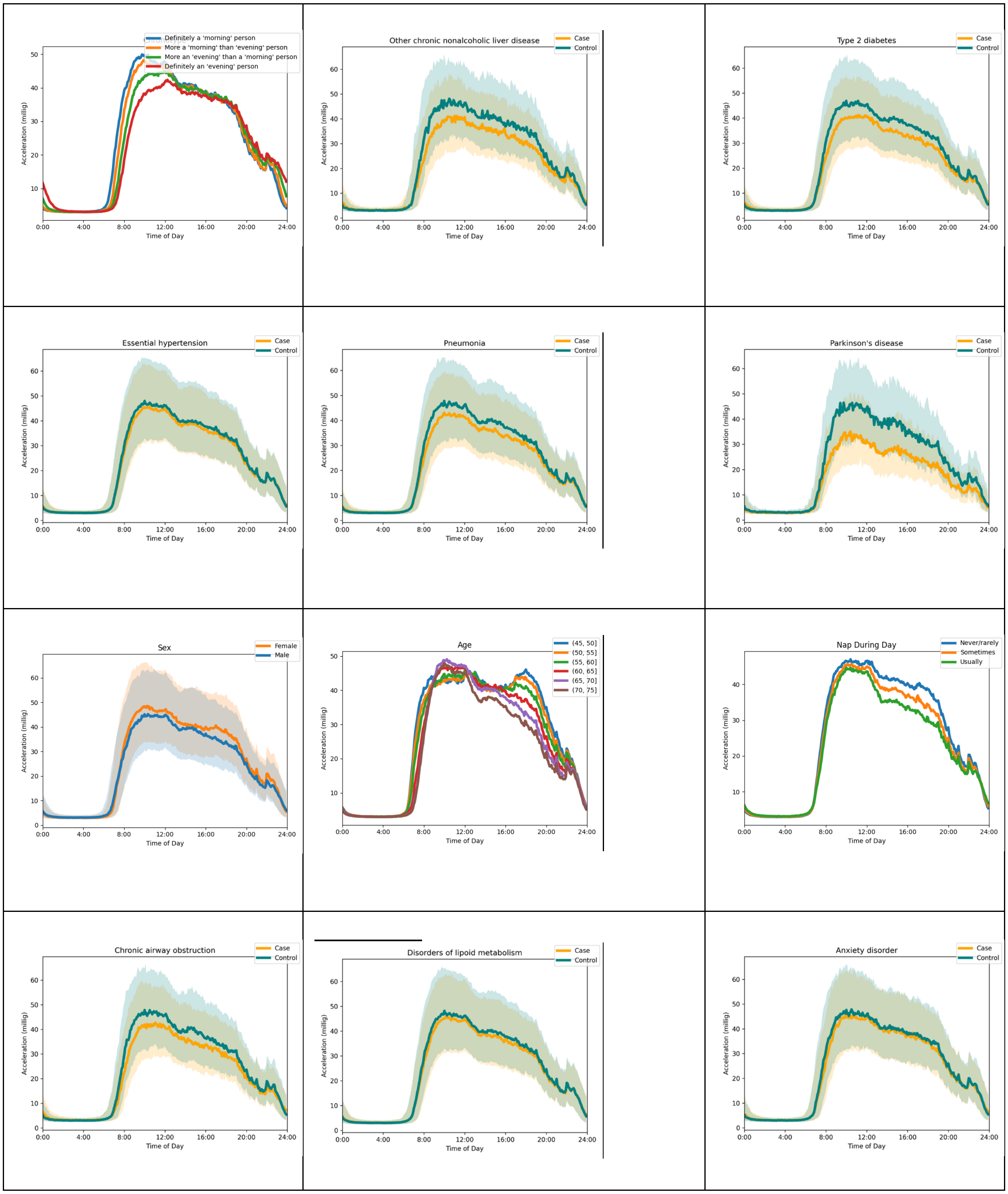
Definitely a 'morning' person
More a 'morning' than 'evening' person
More an 'evening' than a 'morning' person
Definitely an 'evening' person
Other chronic nonalcoholic liver disease
Type 2 diabetes
Essential hypertension
Pneumonia
Parkinson's disease
Sex
Female
Male
Age
(45, 50]
(50, 55]
(55, 60]
(60, 65]
(65, 70]
(70, 75]
Nap During Day
Never/rarely
Sometimes
Usually
Chronic airway obstruction
Disorders of lipoid metabolism
Anxiety disorder
Case
Control
Acceleration (millig)
Time of Day
0:00
4:00
8:00
12:00
16:00
20:00
24:00

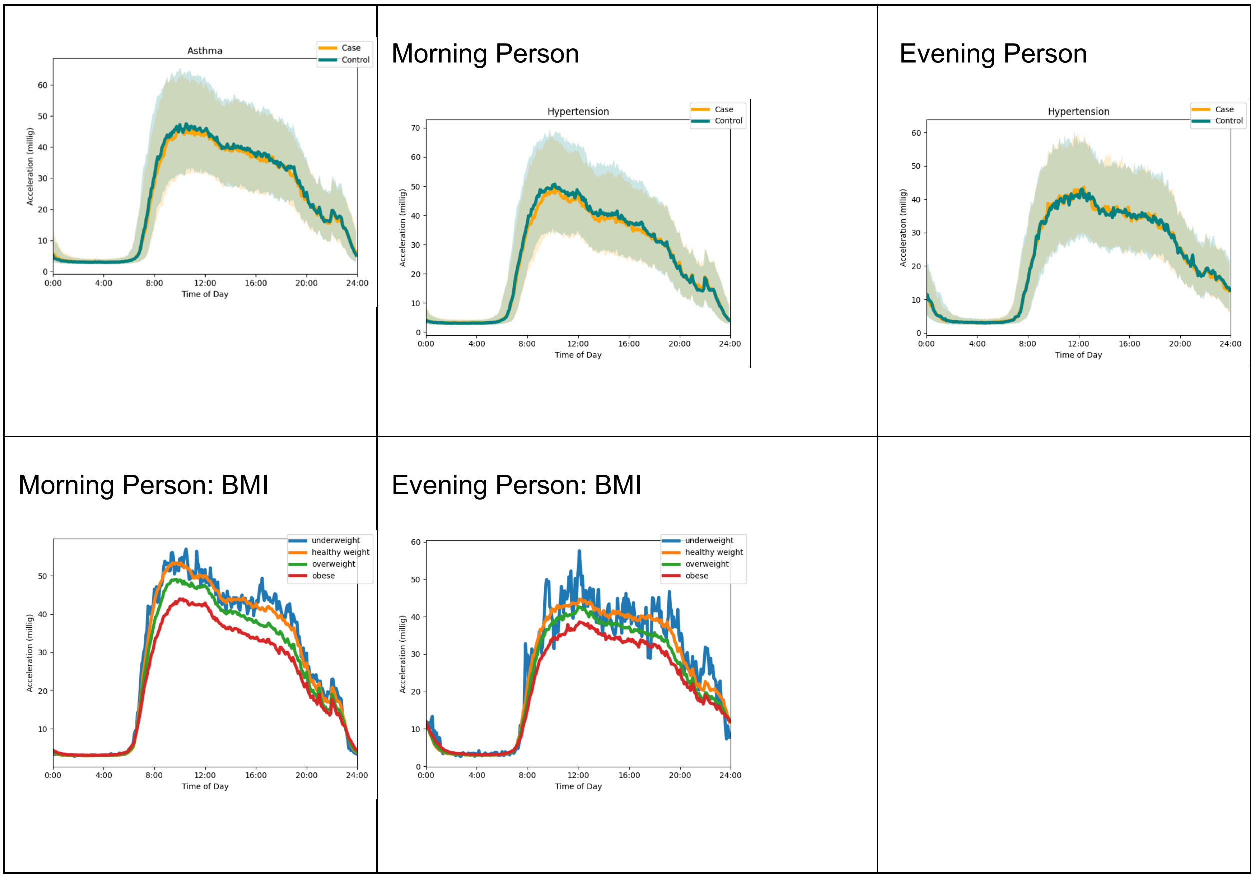


**Supplementary Figure 3 – Acceleration traces**

Median wrist acceleration (vector magnitude after removal of gravitational acceleration) comparing cases and controls and for differences by sex, age, self-reported napping during the day as well as by disease condition for cases with chronic airway obstruction, disorders of lipoid metabolism, anxiety disorder, and asthma. For figures with just two groups, interquartile regions (25$^{th}$ to 75$^{th}$ percentiles) of the population are displayed in shaded regions. See Figure 2 and Figure S2 for descriptions and corresponding temperature plots.

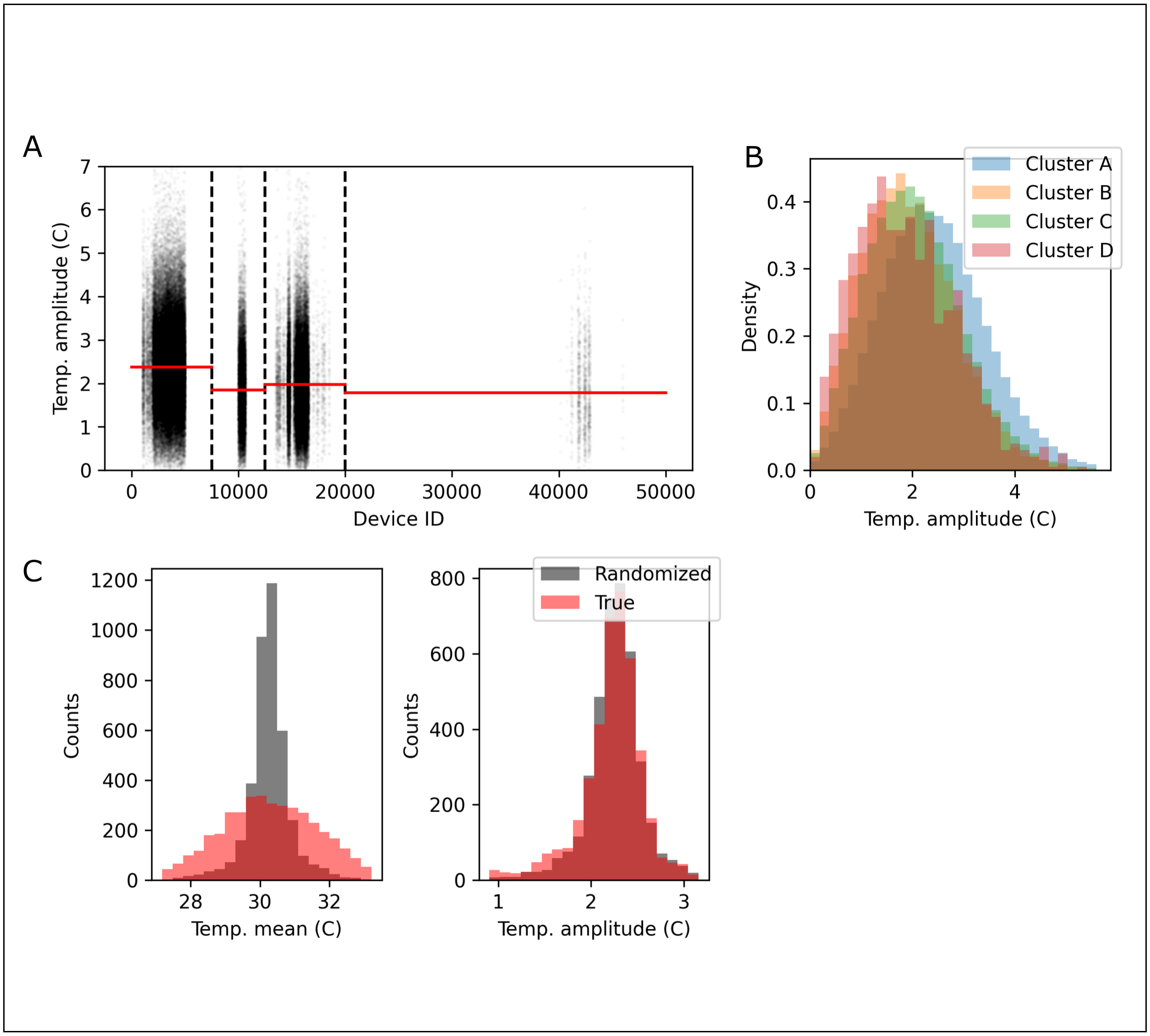


**Supplementary Figure 4 – Calibration of temperature data**

(A) Temperature amplitudes by device ID. Three distinct groups of device IDs are present, divided by the dashed vertical lines. Each cluster demonstrates distinct bias, with the median value of each shown in red. (Cluster A: device IDs up to 7,500, Cluster B: 7,501 through 12,500, Cluster C: 12,501 through 20,000, Cluster D: above 20,000). (B) The distributions of the temperature cosinor amplitude (temp_amplitude) in each of the

clusters of actigraphy devices. Cluster D only contains readings from the repeated actigraphy measurements and so is considerably smaller in size. (C) The distribution of average (bottom left) temperature means and (right) temperature amplitude scores among all measurements from one device, in red, compared to the distribution after a random permutation of device IDs (in grey) to show expected variance. Wider true distributions than by random permutation indicate existence of device-specific biases. While mean temperature shows prominent device-specific bias, temperature rhythms are nearly device-independent.

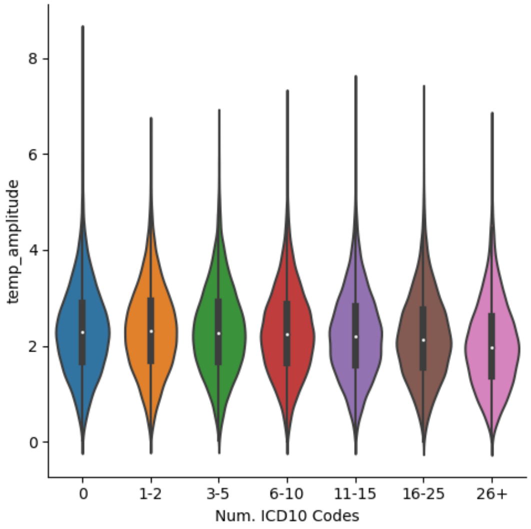


**Supplementary Figure 5 – Stratification by Number of Diagnoses**

All unique ICD-10 diagnoses were counted for each participant irrespective of time of diagnosis. The population was stratified according to the number of distinct diagnoses, to capture a measure of multimorbidity. Shown are temperature amplitudes distributions for each diagnosis counts as violin plots. Box plots show the median (white dot), inter-quartile range (thick black line) and minimum/maximimum (thin line). The number of participants in each group is *n* =13907 (0 codes), *n*=13141 (1-2 codes), *n*=16431(3-5

codes), *n*=18599 (6-10 codes), *n*=11008 (11-15 codes), *n*=10696 (16-25 codes), *n*=8540 (26 or more codes).

## Supplementary Tables

Supplementary Table 1 - Demographics of participants with and without actigraphy in the UK Biobank.

| | With Actigraphy | Without Actigraphy |
|---|---|---|
| **N** | 91,462 | 398,825 |
| **Male** | 43.7% | 46.1% |
| **Female** | 56.3% | 53.9% |
| **White** | 96.6% | 93.4% |
| **Nonwhite** | 3.4% | 6.6% |
| **Birth Year mean (SD)** | 1952.0 (7.8) | 1951.4 (8.2) |
| **Age at Actigraphy Mean (SD)** | 62.8 (7.8) | N/A |
| **BMI mean (SD)** | 26.7 (4.5) | 27.6 (4.9) |

## Supplementary References


1. Doherty A*, et al.* Large Scale Population Assessment of Physical Activity Using Wrist Worn Accelerometers: The UK Biobank Study. *PLoS One* **12**, e0169649 (2017).

2. Willetts M, Hollowell S, Aslett L, Holmes C, Doherty A. Statistical machine learning of sleep and physical activity phenotypes from sensor data in 96,220 UK Biobank participants. *Sci Rep* **8**, 7961 (2018).

3. Lyall LM*, et al.* Association of disrupted circadian rhythmicity with mood disorders, subjective wellbeing, and cognitive function: a cross-sectional study of 91 105 participants from the UK Biobank. *Lancet Psychiatry* **5**, 507-514 (2018).

4. Ferguson A*, et al.* Genome-Wide Association Study of Circadian Rhythmicity in 71,500 UK Biobank Participants and Polygenic Association with Mood Instability. *EBioMedicine* **35**, 279-287 (2018).

5. Kennard HR, Huebner GM, Shipworth D, Oreszczyn T. The associations between thermal variety and health: Implications for space heating energy use. *PLoS One* **15**, e0236116 (2020).

6. Fry A*, et al.* Comparison of Sociodemographic and Health-Related Characteristics of UK Biobank Participants With Those of the General Population. *Am J Epidemiol* **186**, 1026-1034 (2017).

7. Shrout PE, Fleiss JL. Intraclass correlations: uses in assessing rater reliability. *Psychol Bull* **86**, 420-428 (1979).
