# Supplementary material for: Diurnal rhythms of wrist temperature are associated with future disease risk in the UK Biobank
Source: Nat Commun. 2023 Aug 24;14:5172. doi: 10.1038/s41467-023-40977-5 (PMC10449859; doi:10.1038/s41467-023-40977-5)
Supplement: Supplementary file 2 — Description of Additional Supplementary Files [file 41467_2023_40977_MOESM2_ESM.pdf]

Description of Additional Supplementary Files

Supplementary Data 1

Full results of proportional hazard models for hazard ratios of wrist temperature amplitude, also including age and sex-specific effects, for all phenotypes tested.

Supplementary Data 2

Document showing all plots and results from the alternative models and assessments run on top PheCODEs, see Methods. This includes assessments for validity of the Cox proportional hazards models, inclusion of time-dependent effects, inclusion of non-linear effects, and competing outcomes with death and diagnoses as two possible outcomes. These are performed in a select set of phenotypes to determine validity of the models used here.

Supplementary Data 3

Zip file containing the results of the same analyses as in (Supplementary Data 1) when run using sub cohorts A and B+C instead of the full cohort.
