# Supplementary material for: Diurnal rhythms of wrist temperature are associated with future disease risk in the UK Biobank
Source: Nat Commun. 2023 Aug 24;14:5172. doi: 10.1038/s41467-023-40977-5 (PMC10449859; doi:10.1038/s41467-023-40977-5)
Supplement: Supplementary file 4 — Supplementary Data 2 [file 41467_2023_40977_MOESM4_ESM.docx]

**Supplementary Data 2**

### Diurnal rhythms of wrist temperature are associated with future disease risk in the UK Biobank

**Thomas G. Brooks PhD**^1*^**, Nicholas F. Lahens PhD**^1^**, Gregory R. Grant PhD**^1,2^**, Yvette I. Sheline MD**^3,4,5^**, Garret A. FitzGerald MD**^1,6^**, & Carsten Skarke MD**^1,6*^

^1^ Institute for Translational Medicine and Therapeutics (ITMAT), ^2^ Department of Genetics, ^3^Department of Radiology, ^4^Department of Psychiatry, ^5^Department of Neurology, ^6^Department of Medicine, University of Pennsylvania Perelman School of Medicine, Philadelphia, PA, USA

*Corresponding Authors:

Thomas G. Brooks, PhD ([thobr@sas.upenn.edu](mailto:thobr@sas.upenn.edu)) & Carsten Skarke, MD ([cskarke@pennmedicine.upenn.edu](mailto:cskarke@pennmedicine.upenn.edu)) Institute for Translational Medicine and Therapeutics (ITMAT), University of Pennsylvania Perelman School of Medicine, Smilow Center for Translational Research 10-101, 3400 Civic Center Blvd, Philadelphia, Pennsylvania 19104, USA

# Cox Proportional Hazards Diagnostics

This document contains supplemental tests performed to asses the impact of various assumptions in the Cox proportional hazards models. These assumptions are the proportional hazards assumption (that hazard ratios are constant over time) and the linearity of the effect. We also assess a competing outcomes models, where death is considered as a competing outcome rather than as a censoring.

This document is large and we recommend navigating by the headings (enable View -> Navigation Pane if using MS Word or similar in other document viewers).

## Proportional Hazards Assumption

We assess the proprtional hazards assumption through the use of the R package 'survival' and it's function called 'cox.zph'. This examines the Schoenfeld residuals. The Schoenfeld residuals 'can essentially be thought of as the observed minus the expected values of the covariates at each failure time' (Steffensmeier & Jones, 2004: p 121).

Below, we plot for a selection of our top phenotypes, the Schoenfeld residual for each particiapnt in the analysis in black, against the time (in years) since the actigraphy measurement was taken. In red, a fit curve for the residuals. If the proportional hazards model is violated, then this curve will be non-horizontal. This is done for each of the covariates in the model.

For each, we also give the p-value from cox.zph of a deviation from horizontal. Due to the high number of subjects, we consider a p < 0.01 threshold for significance and add a star (*) to those p-values.

### Type 2 diabetes

Type 2 diabetes - sex - p = 0.651


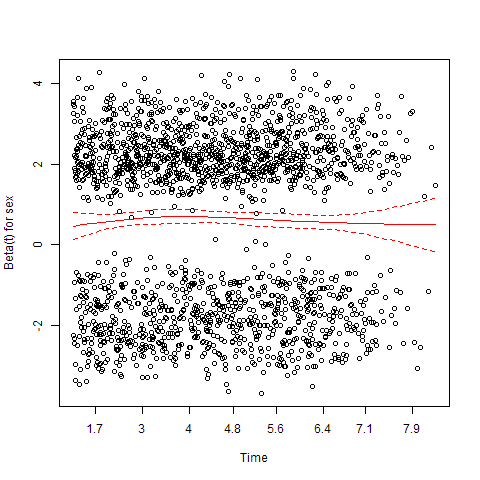


Type 2 diabetes - ethnicity_white - p = 0.761


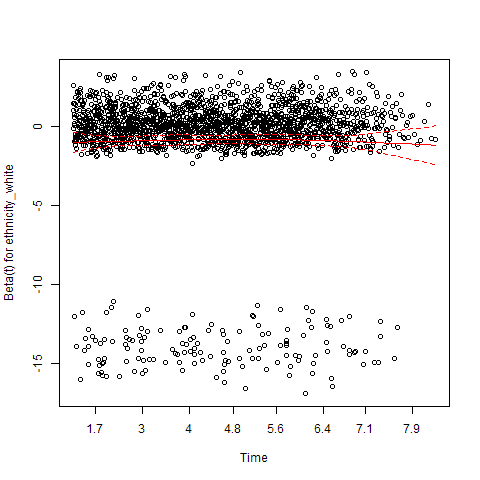


Type 2 diabetes - overall_health - p = 0.249


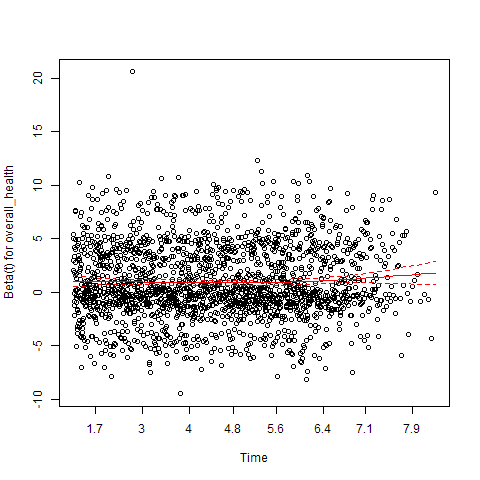


Type 2 diabetes - smoking - p = 0.142


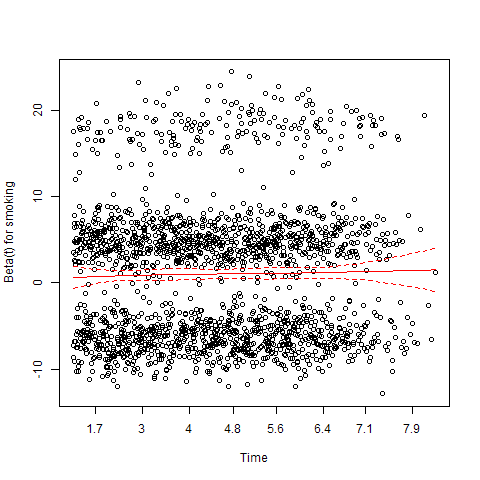


Type 2 diabetes - age_at_actigraphy_cat - p = 0.320


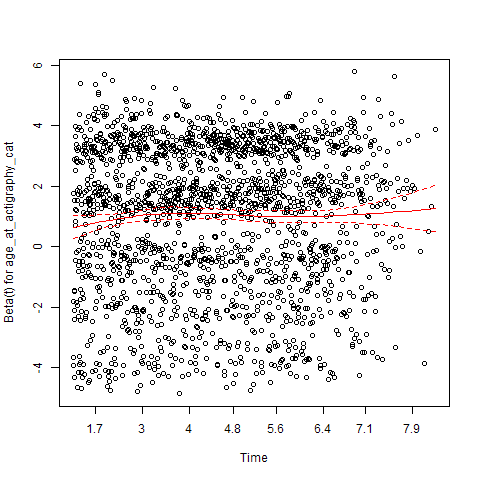


Type 2 diabetes - BMI - p = 0.645


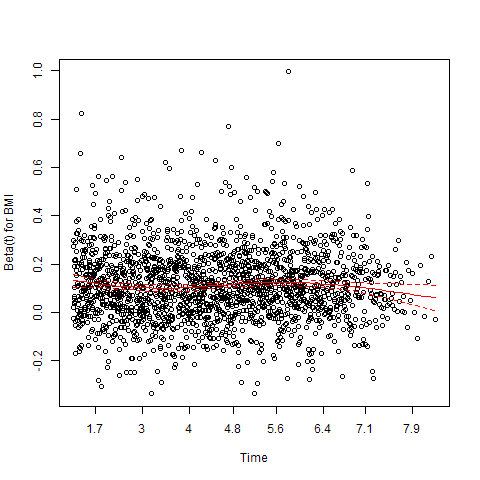


Type 2 diabetes - college_education - p = 0.963


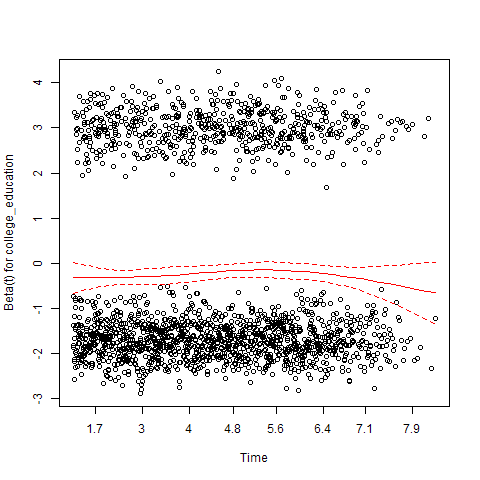


Type 2 diabetes - alcohol_frequency - p = 0.107


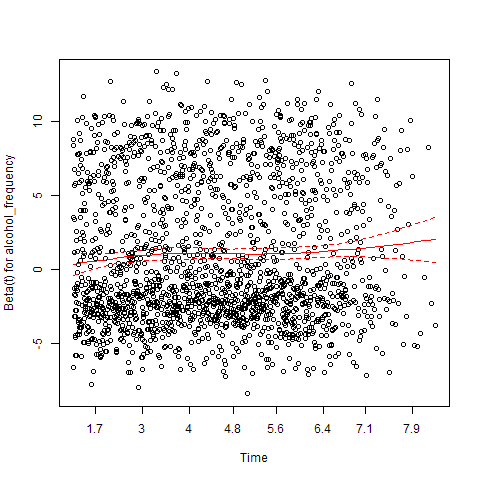


Type 2 diabetes - townsend_deprivation_index - p = 0.156


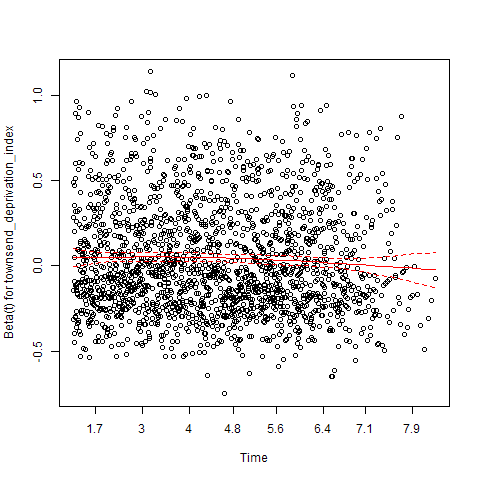


### Essential hypertension

Essential hypertension - sex - p = 0.063


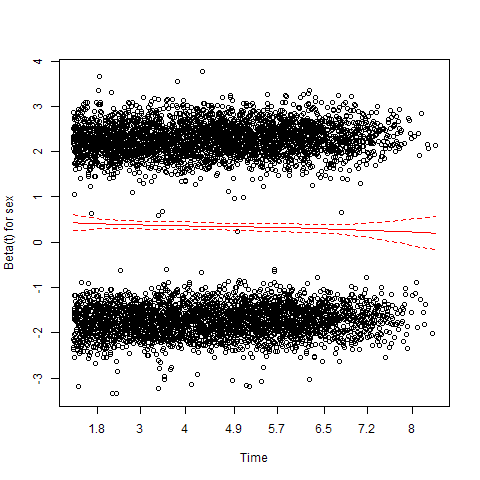


Essential hypertension - ethnicity_white - p = 0.046


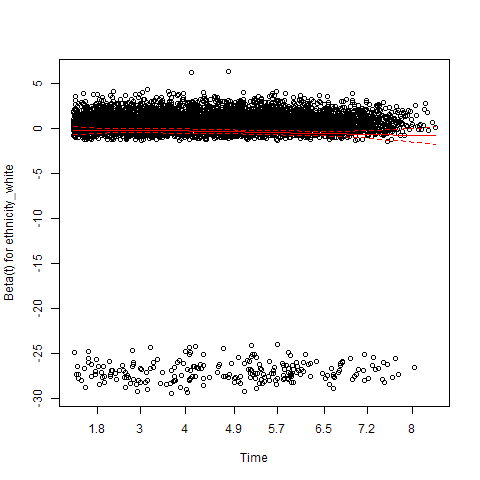


Essential hypertension - overall_health - p = 0.288


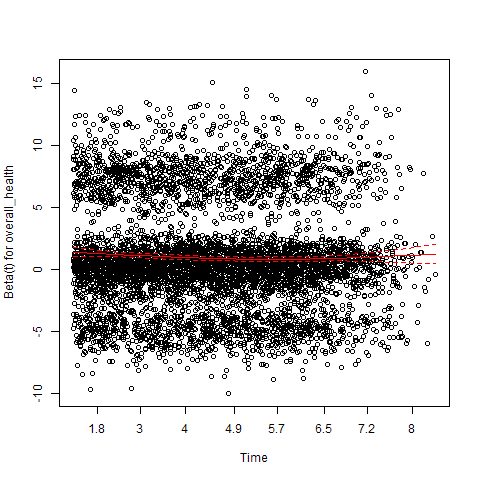


Essential hypertension - smoking - p = 0.190


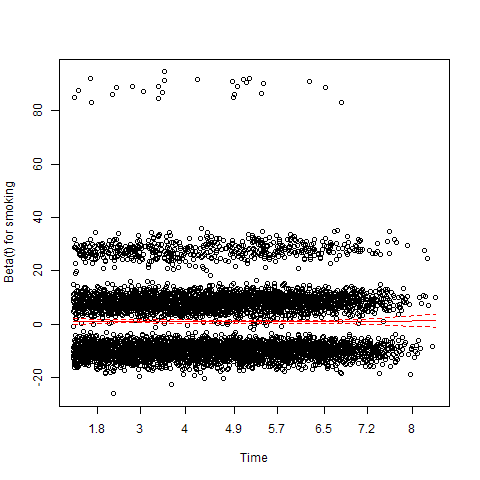


Essential hypertension - age_at_actigraphy_cat - p = 0.647


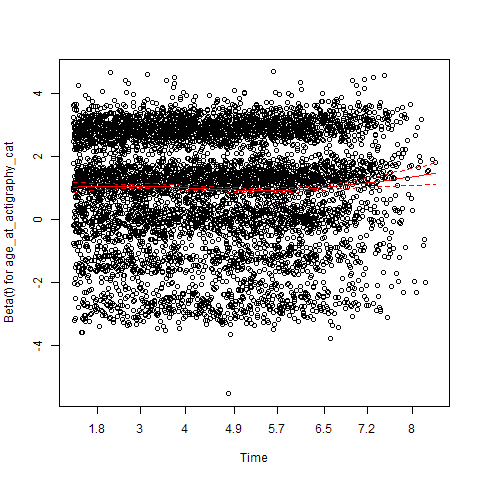


Essential hypertension - BMI - p = 0.166


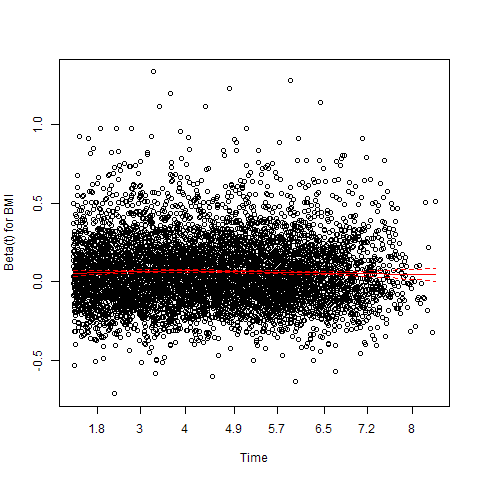


Essential hypertension - college_education - p = 0.518


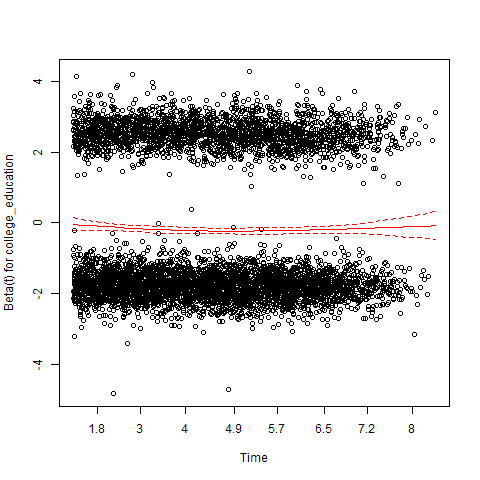


Essential hypertension - alcohol_frequency - p = 0.427


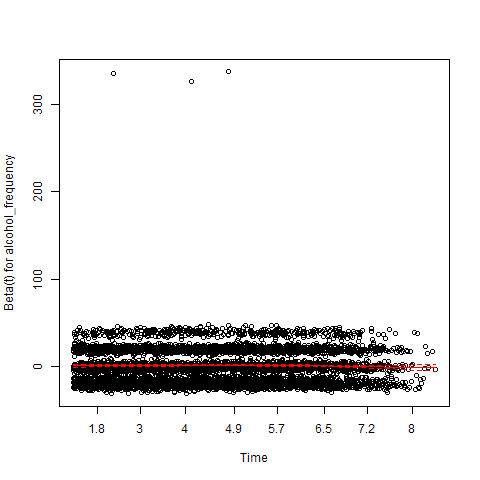


Essential hypertension - townsend_deprivation_index - p = 0.084


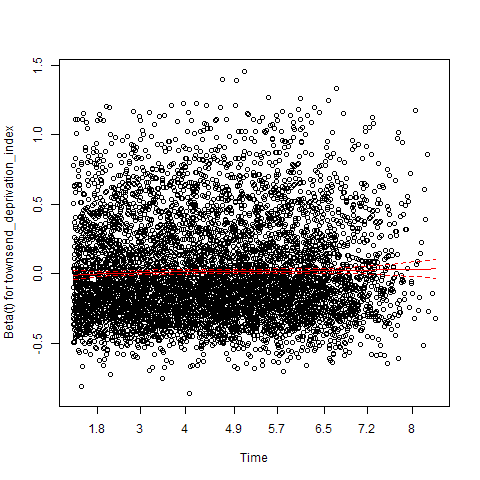


### Other chronic nonalcoholic liver disease

### Renal failure

Renal failure - sex - p = 0.073


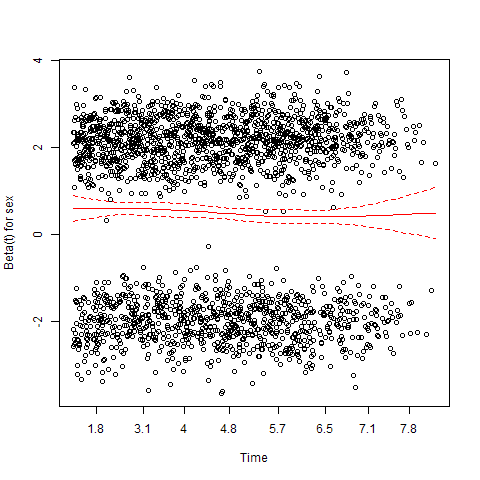


Renal failure - ethnicity_white - p = 0.170


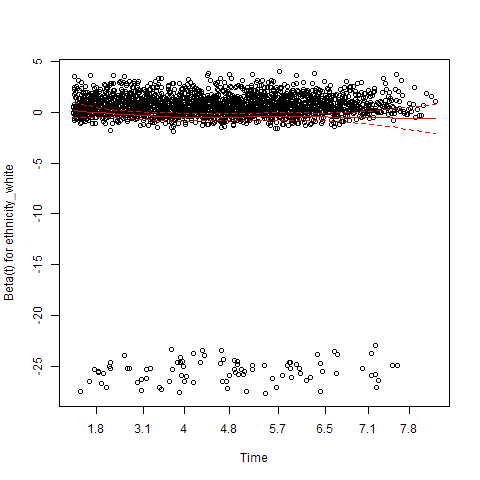


Renal failure - overall_health - p = 0.719


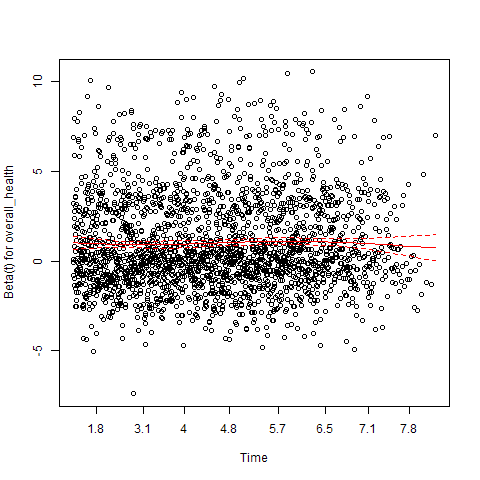


Renal failure - smoking - p = 0.291


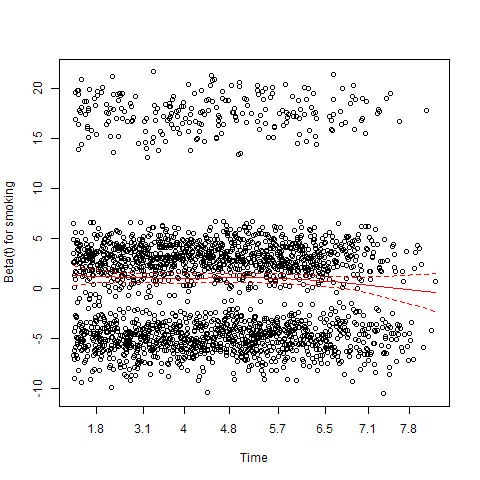


Renal failure - age_at_actigraphy_cat - p = 0.543


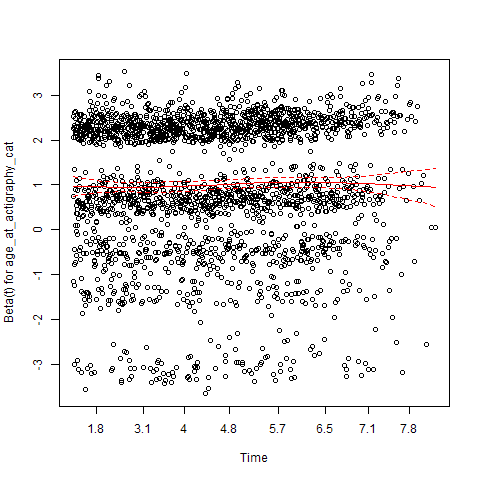


Renal failure - BMI - p = 0.404


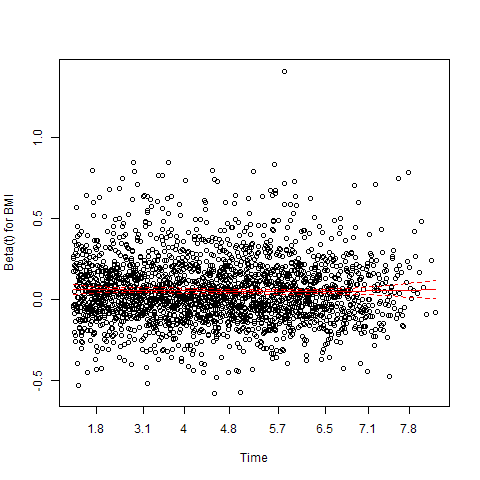


Renal failure - college_education - p = 0.795


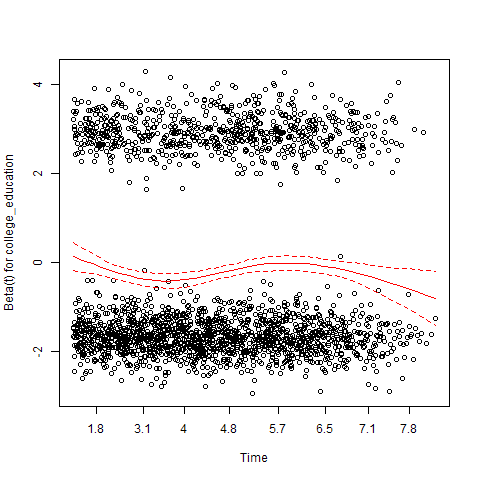


Renal failure - alcohol_frequency - p = 0.724


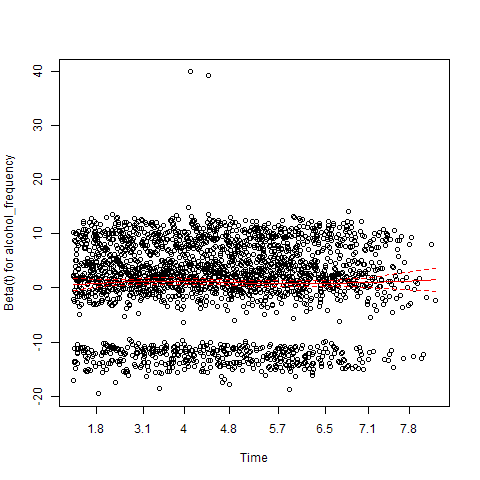


Renal failure - townsend_deprivation_index - p = 0.220


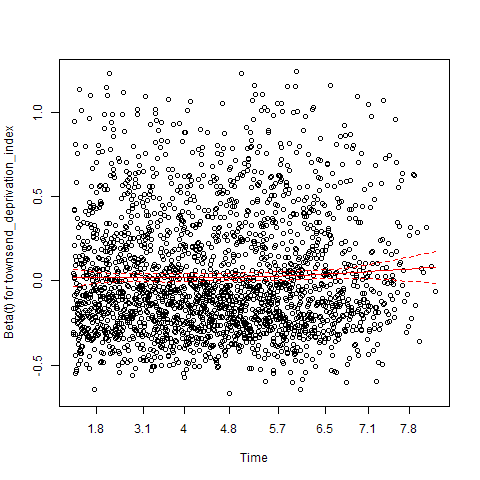


### Diverticulosis

Diverticulosis - sex - p = 0.241


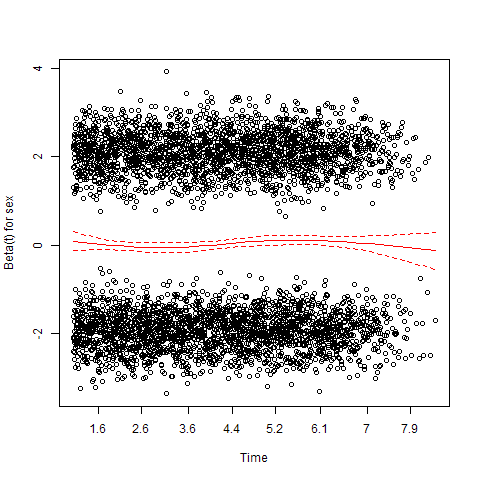


Diverticulosis - ethnicity_white - p = 0.427


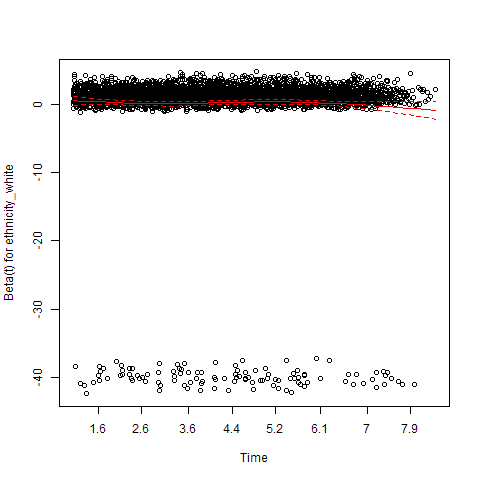


Diverticulosis - overall_health - p = 0.846


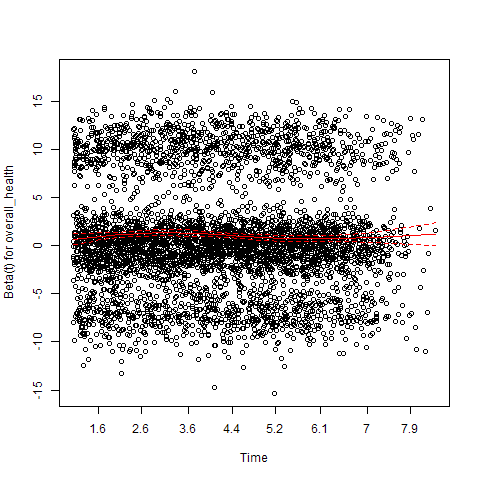


Diverticulosis - smoking - p = 0.859


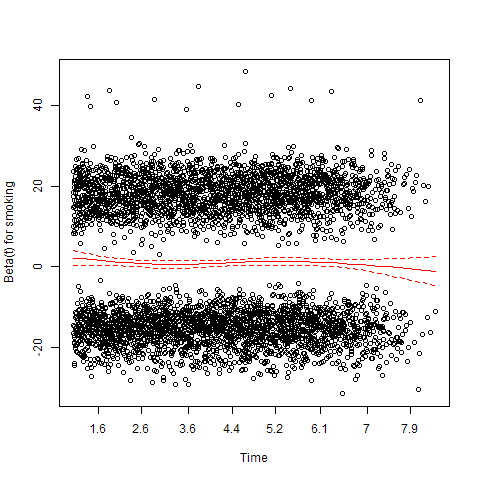


Diverticulosis - age_at_actigraphy_cat - p = 0.024


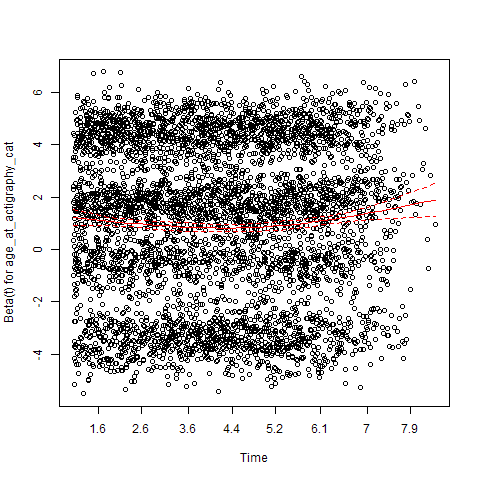


Diverticulosis - BMI - p = 0.651


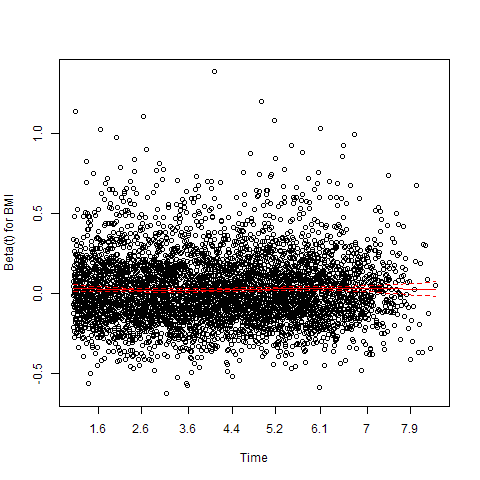


Diverticulosis - college_education - p = 0.324


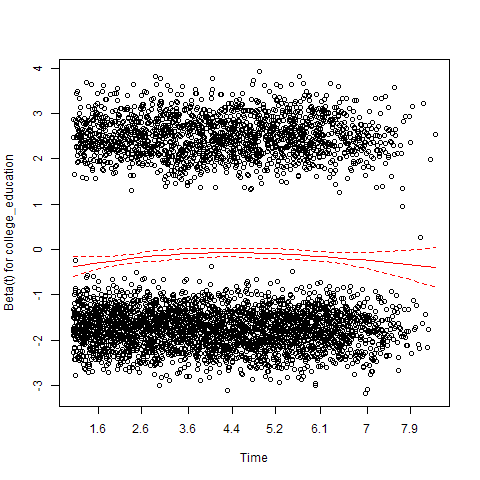


Diverticulosis - alcohol_frequency - p = 0.290


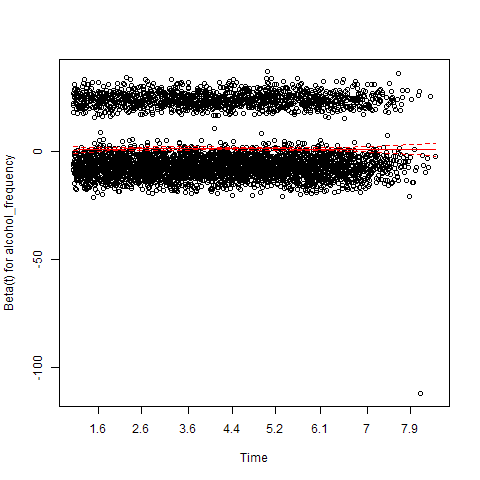


Diverticulosis - townsend_deprivation_index - p = 0.746


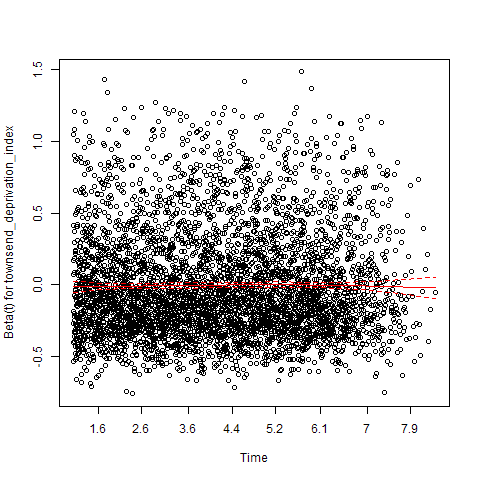


### Pneumonia

Pneumonia - sex - p = 0.148


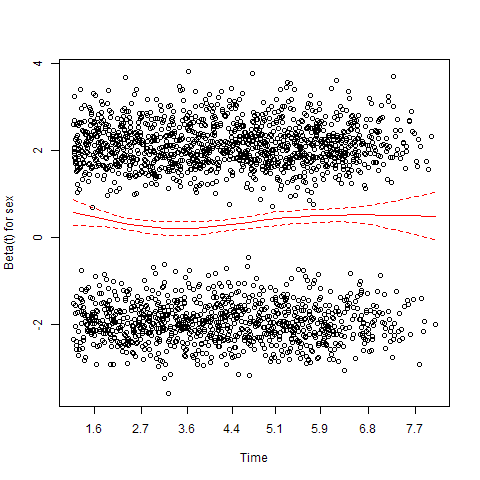


Pneumonia - ethnicity_white - p = 0.518


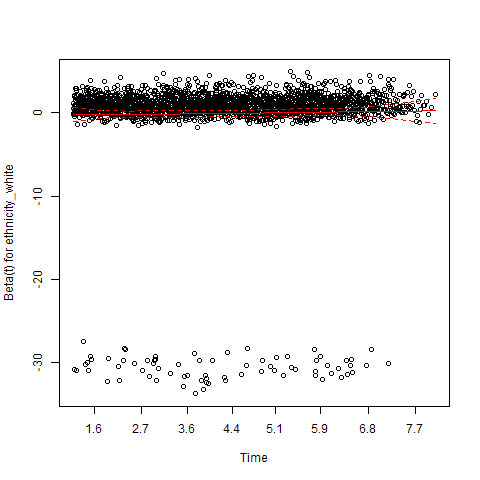


Pneumonia - overall_health - p = 0.754


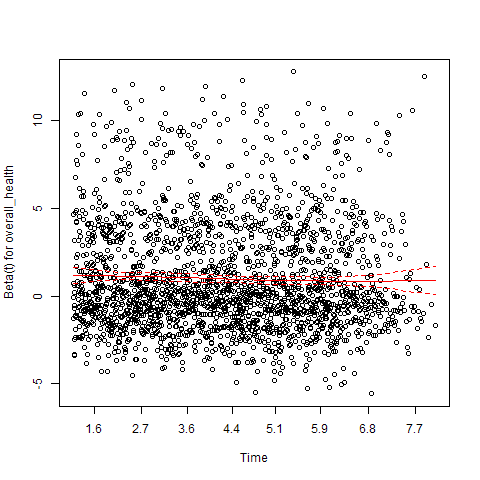


Pneumonia - smoking - p = 0.030


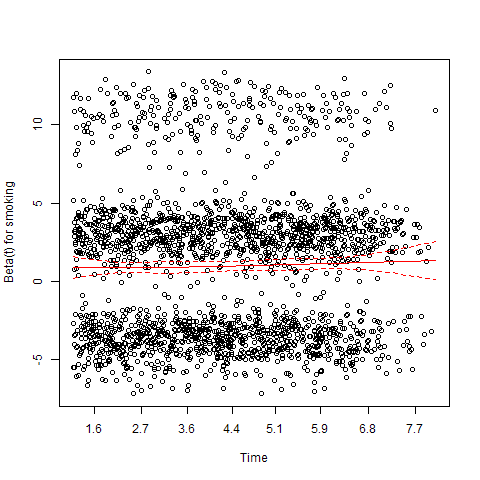


Pneumonia - age_at_actigraphy_cat - p = 0.024


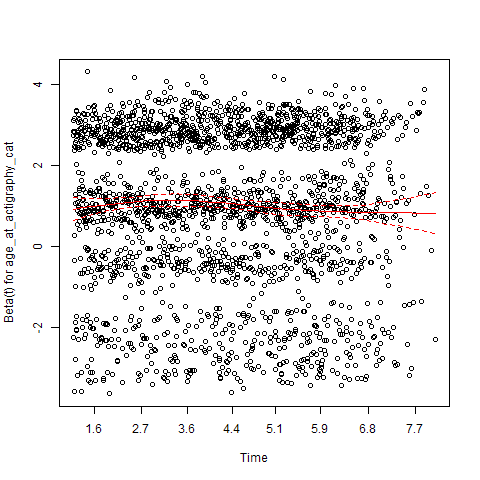


Pneumonia - BMI - **p = 0.001***


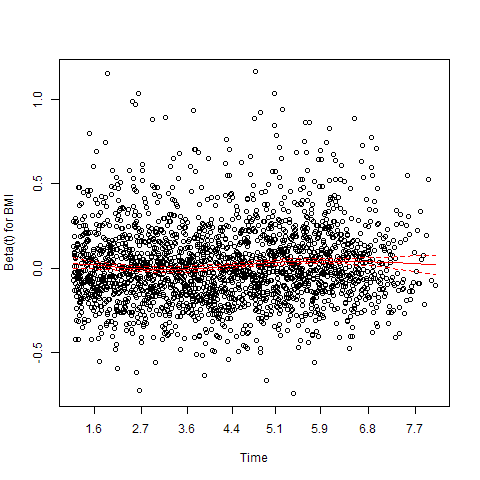


Pneumonia - college_education - p = 0.450


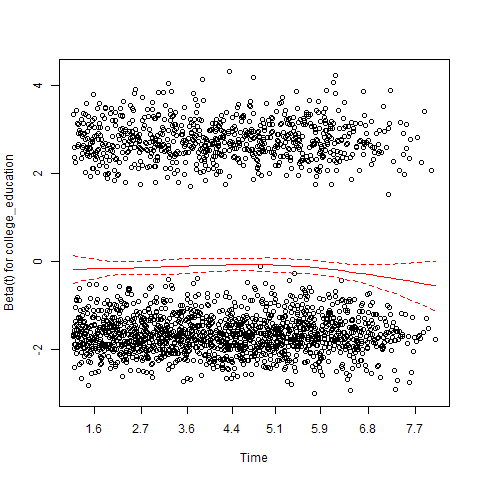


Pneumonia - alcohol_frequency - p = 0.882


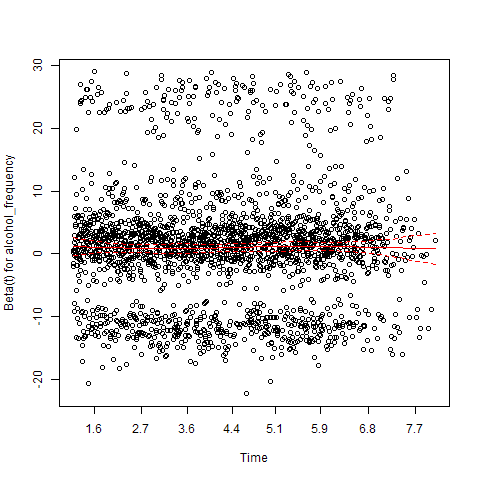


Pneumonia - townsend_deprivation_index - p = 0.067


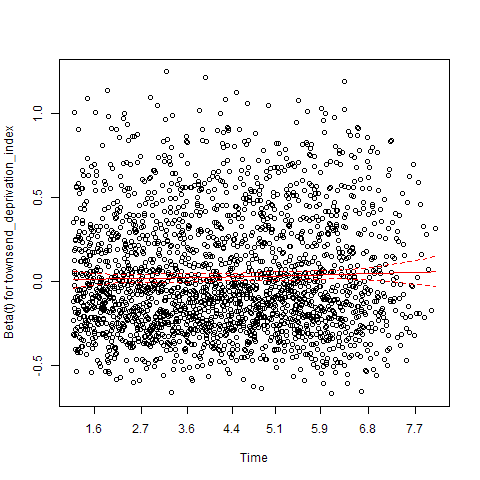


### Disorders of lipoid metabolism

Disorders of lipoid metabolism - sex - p = 0.958


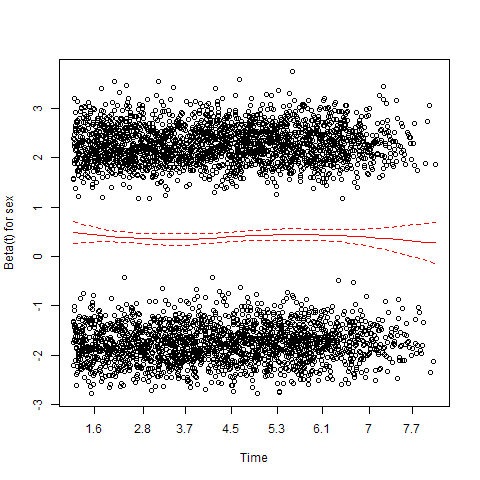


Disorders of lipoid metabolism - ethnicity_white - p = 0.353


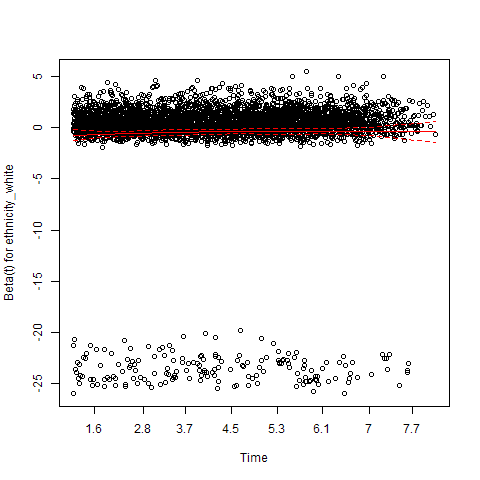


Disorders of lipoid metabolism - overall_health - p = 0.840


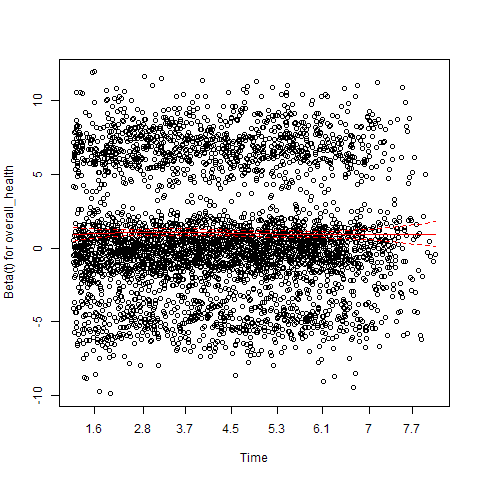


Disorders of lipoid metabolism - smoking - p = 0.839


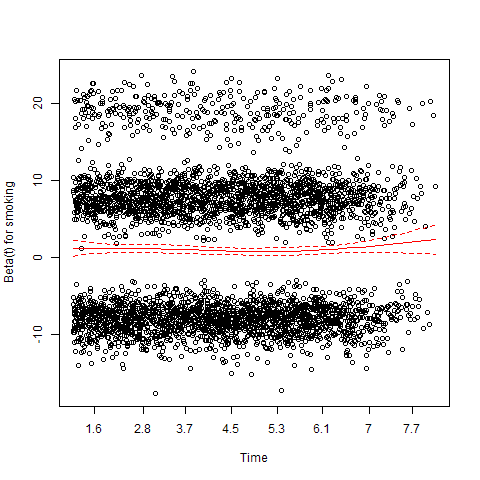


Disorders of lipoid metabolism - age_at_actigraphy_cat - p = 0.429


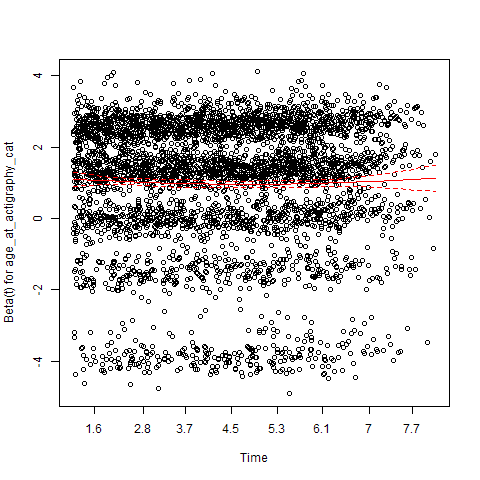


Disorders of lipoid metabolism - BMI - p = 0.041


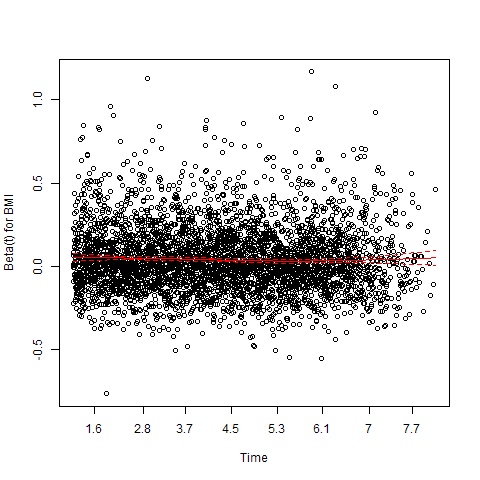


Disorders of lipoid metabolism - college_education - p = 0.226


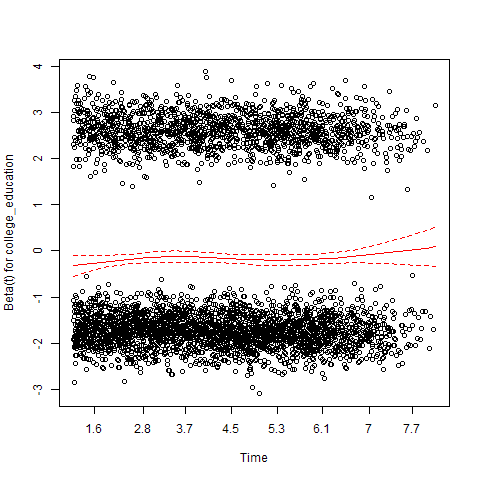


Disorders of lipoid metabolism - alcohol_frequency - p = 0.719


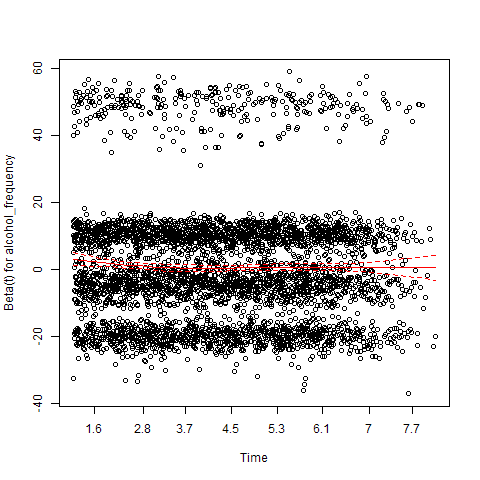


Disorders of lipoid metabolism - townsend_deprivation_index - **p = 0.000***


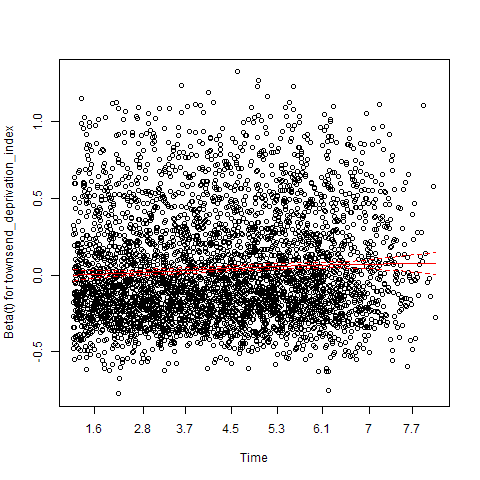


### Sleep disorders

### Osteoarthrosis

Osteoarthrosis - sex - p = 0.646


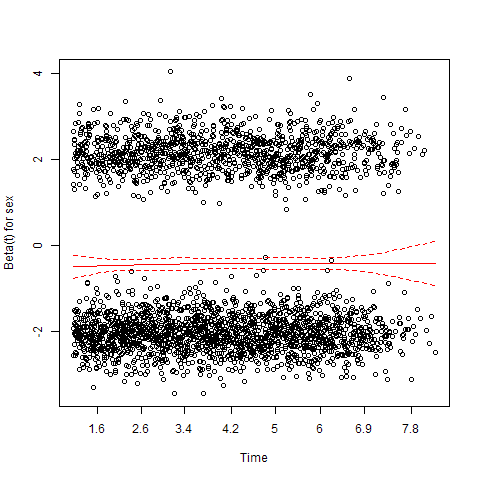


Osteoarthrosis - ethnicity_white - p = 0.595


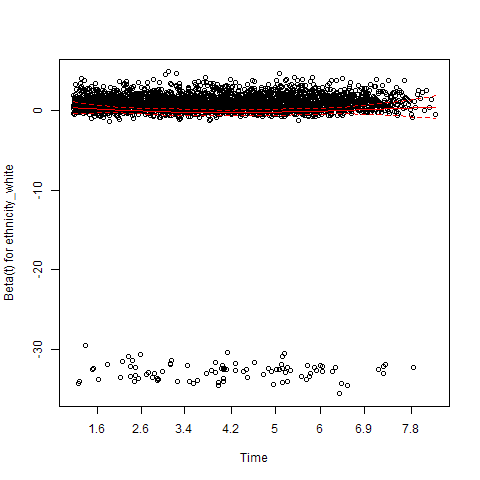


Osteoarthrosis - overall_health - p = 0.647


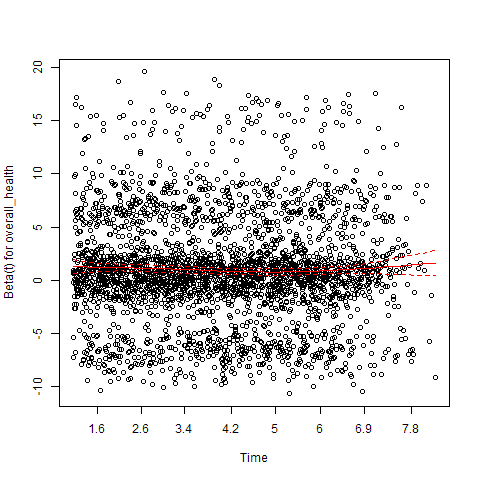


Osteoarthrosis - smoking - p = 0.780


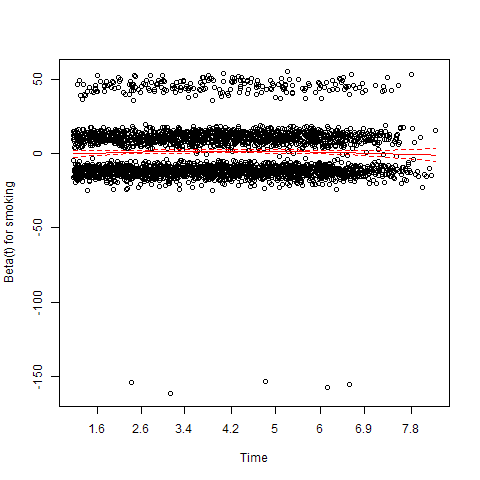


Osteoarthrosis - age_at_actigraphy_cat - p = 0.644


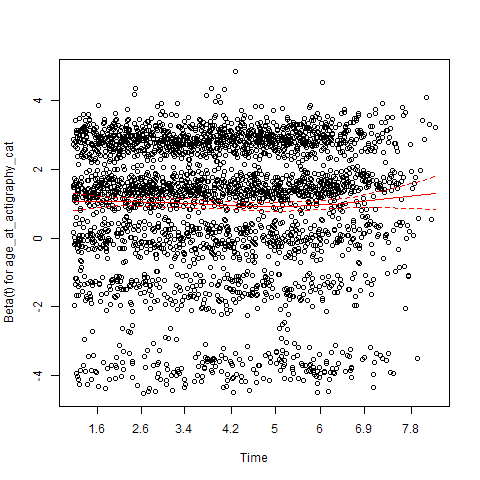


Osteoarthrosis - BMI - p = 0.432


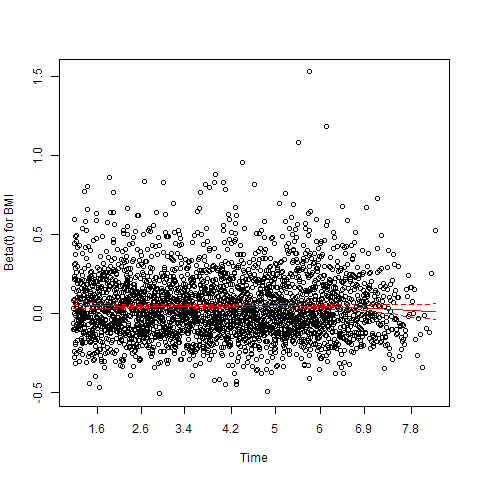


Osteoarthrosis - college_education - p = 0.690


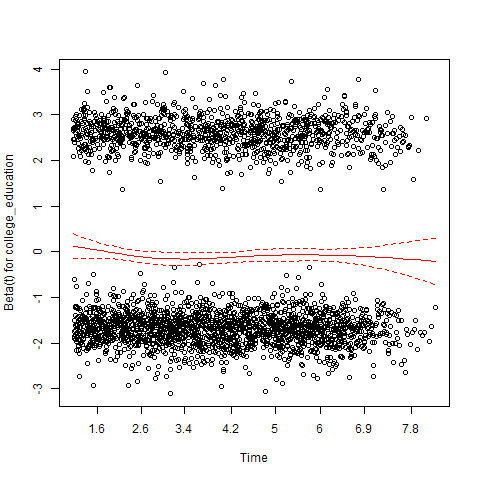


Osteoarthrosis - alcohol_frequency - p = 0.179


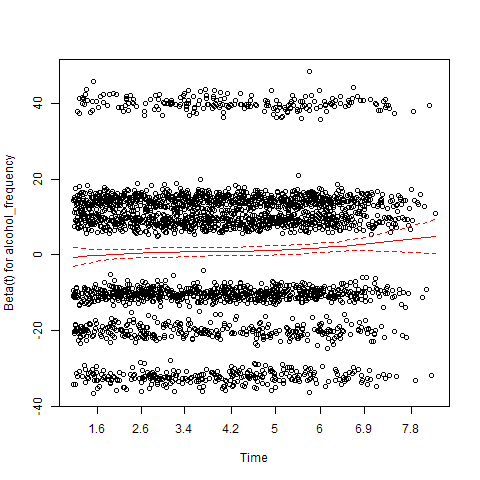


Osteoarthrosis - townsend_deprivation_index - p = 0.140


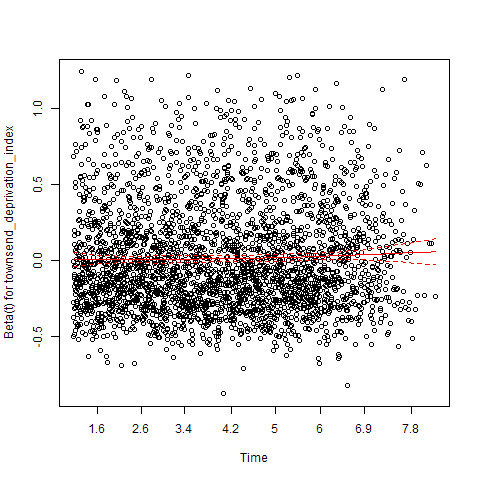


### Extrapyramidal disease and abnormal movement disorders

### Anxiety disorders

Anxiety disorders - sex - **p = 0.006***


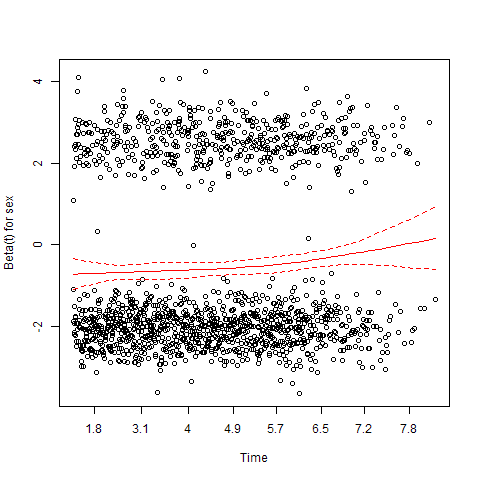


Anxiety disorders - ethnicity_white - p = 0.576


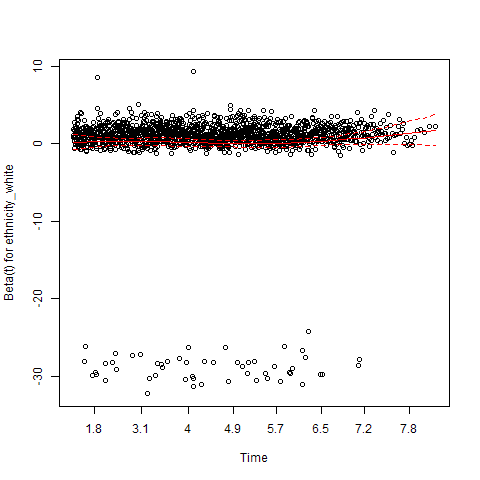


Anxiety disorders - overall_health - p = 0.805


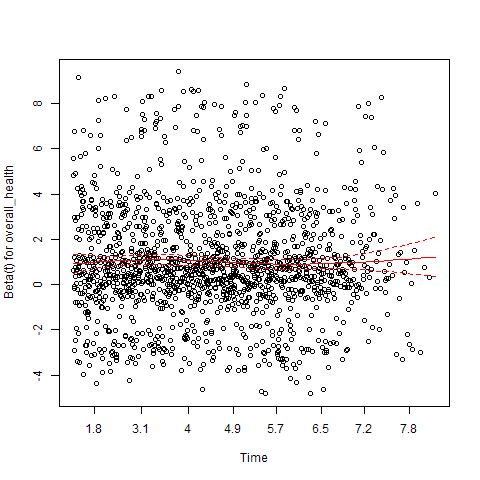


Anxiety disorders - smoking - p = 0.274


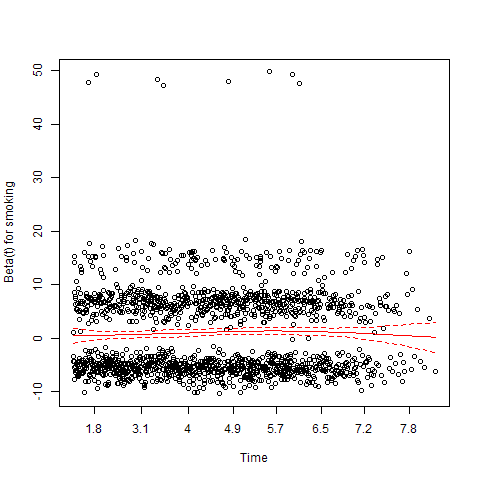


Anxiety disorders - age_at_actigraphy_cat - p = 0.453


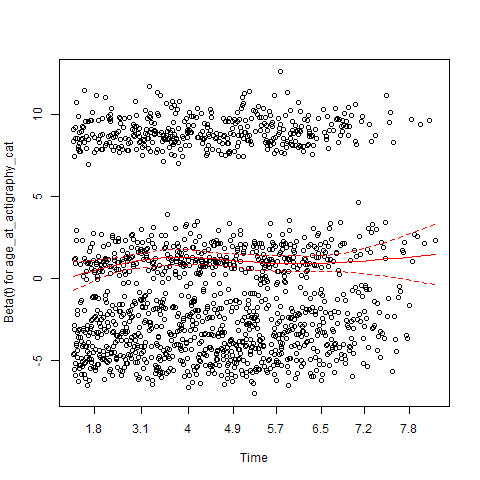


Anxiety disorders - BMI - p = 0.927


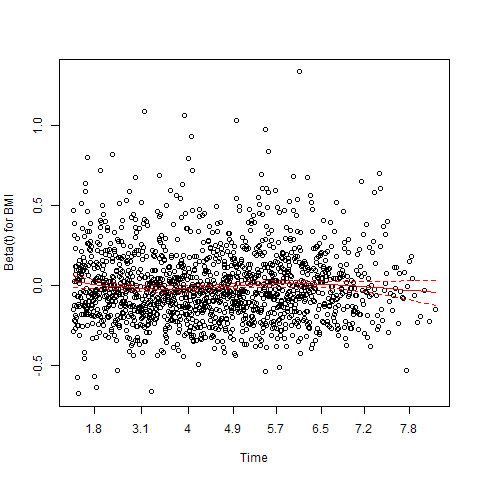


Anxiety disorders - college_education - p = 0.748


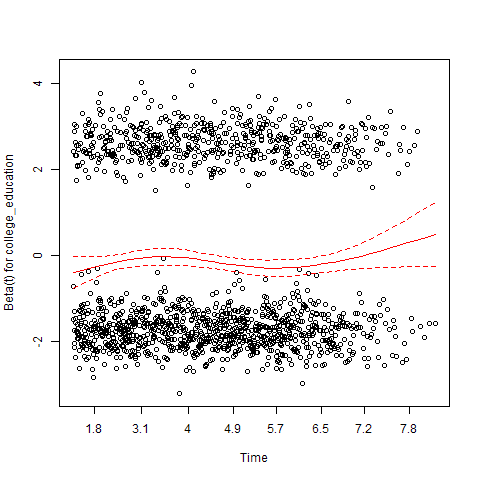


Anxiety disorders - alcohol_frequency - p = 0.813


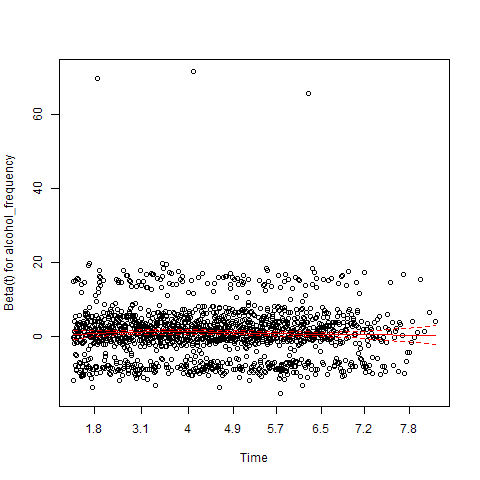


Anxiety disorders - townsend_deprivation_index - p = 0.333


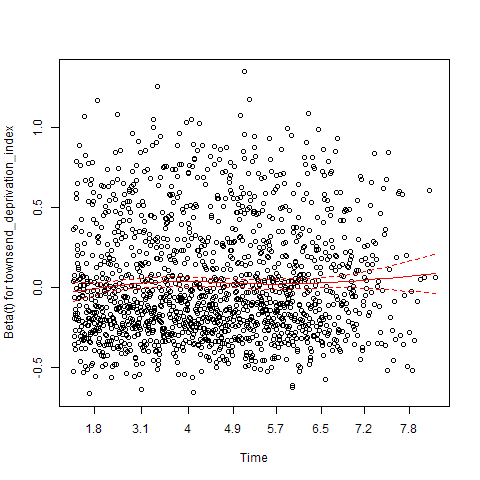


### Parkinson's disease

## Time-varying Effects

Based off the identified significant deviations from the Cox proportional hazards model, we next introduce models that include time-varying effects for all the identified covariates that violate the assumptions. This corrects for the deviations from the constant hazard ratios assumption.

The results of running each of these models is displayed below. The table displays the p-value and effect size of the temperature amplitude effect. These can be compared to the main results in the manuscript for inconsistent results.

|  | p | logHR | logHR_se | std_logHR | std_logHR_se |
| --- | --- | --- | --- | --- | --- |
| Type 2 diabetes | 6.9123e-23 | -2.8963e-01 | 2.9406e-02 | -2.6372e-01 | 2.6775e-02 |
| Essential hypertension | 6.7476e-14 | -1.1531e-01 | 1.5389e-02 | -1.0499e-01 | 1.4012e-02 |
| Renal failure | 2.6838e-06 | -1.2117e-01 | 2.5815e-02 | -1.1033e-01 | 2.3505e-02 |
| Diverticulosis | 1.2246e-08 | -9.9938e-02 | 1.7544e-02 | -9.0997e-02 | 1.5975e-02 |
| Pneumonia | 2.8985e-05 | -1.0810e-01 | 2.5852e-02 | -9.8424e-02 | 2.3539e-02 |
| Disorders of lipoid metabolism | 5.9246e-06 | -8.3832e-02 | 1.8510e-02 | -7.6332e-02 | 1.6854e-02 |
| Osteoarthrosis | 4.5994e-03 | -6.2649e-02 | 2.2107e-02 | -5.7044e-02 | 2.0130e-02 |
| Anxiety disorders | 6.2957e-02 | -5.6569e-02 | 3.0422e-02 | -5.1508e-02 | 2.7700e-02 |

## Non-linear Effects

We next assess whether the temperature amplitude variable may have a non-linear relation with the hazard ratio for each of the phenotypes. To do this, we use a spline fit (specifically, a pspline with df=3).

Below, we plot each nonlinear model by the HR versus temperature amplitude along with the confidence interval. We note apparent non-linearities that occur outside of the typical range of temperature amplitude values (0.62-4.2 degrees C for the 2.5th-97.5th percentiles) are generally unimportant and nonsignificant.

### Type 2 diabetes


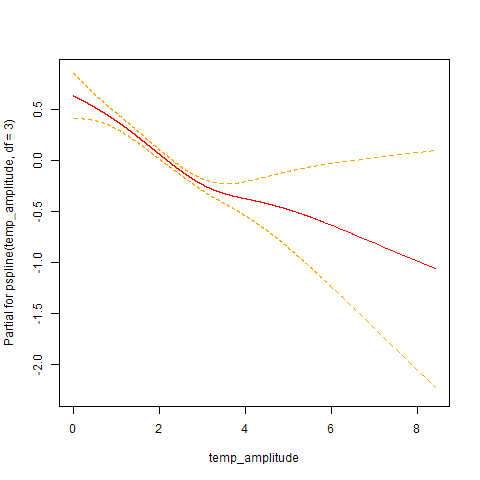


### Essential hypertension


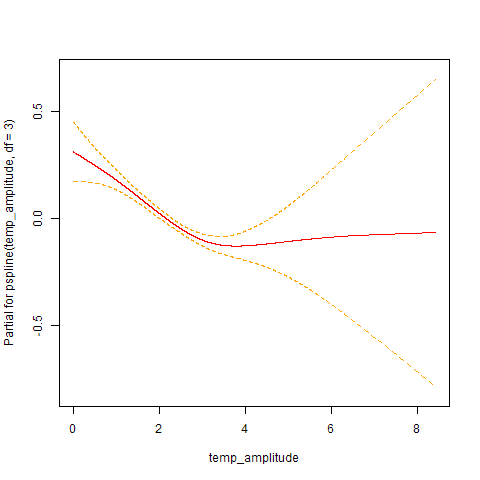


### Other chronic nonalcoholic liver disease


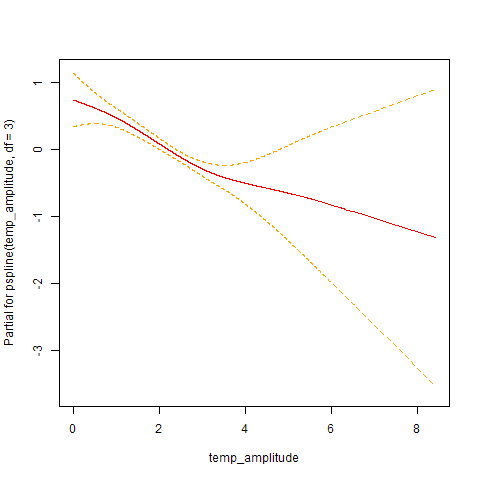


### Renal failure


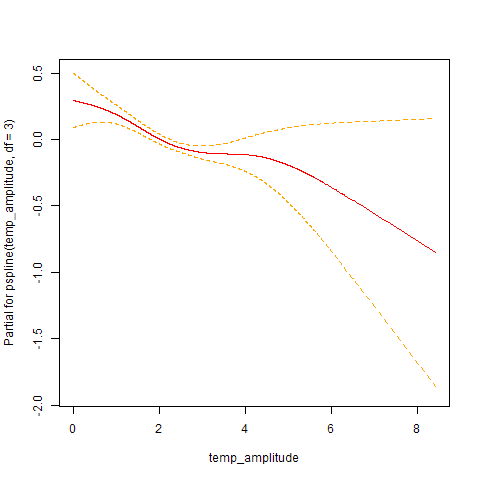


### Diverticulosis


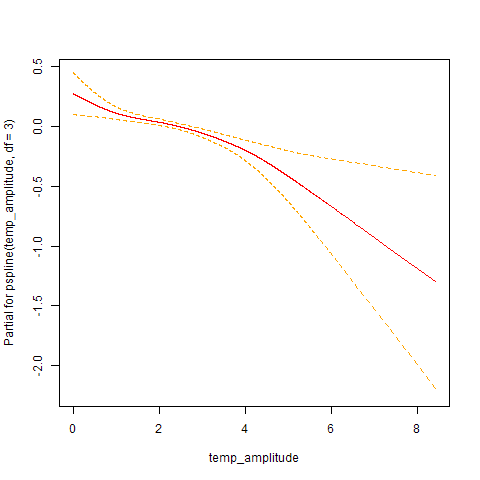


### Pneumonia


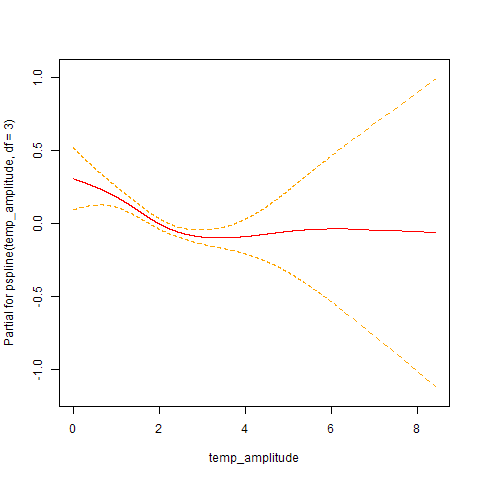


### Disorders of lipoid metabolism


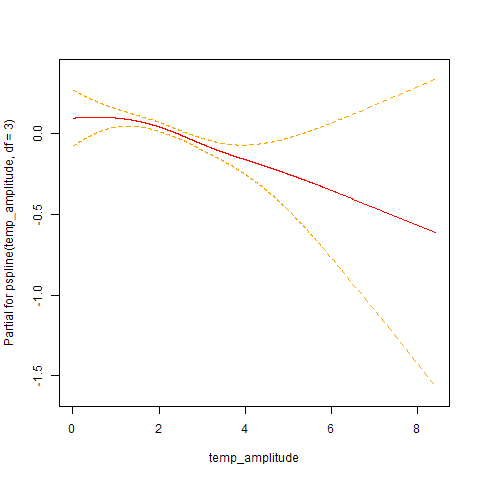


### Sleep disorders


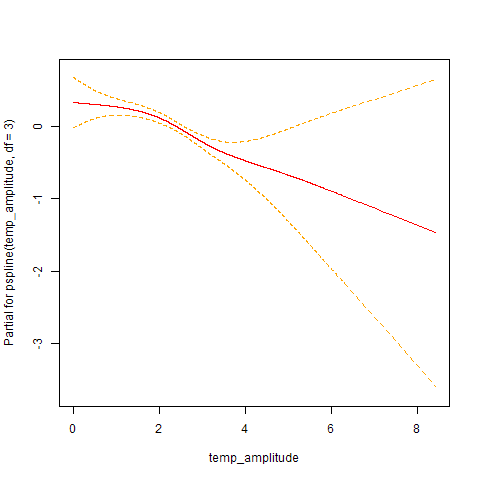


### Osteoarthrosis


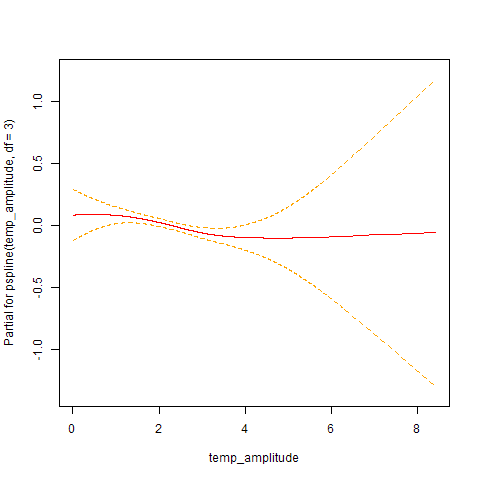


### Extrapyramidal disease and abnormal movement disorders


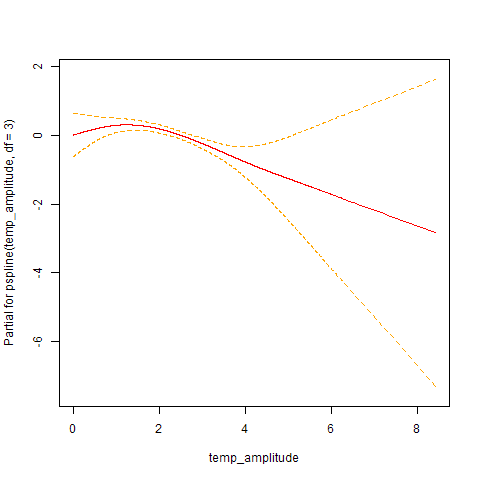


### Anxiety disorders


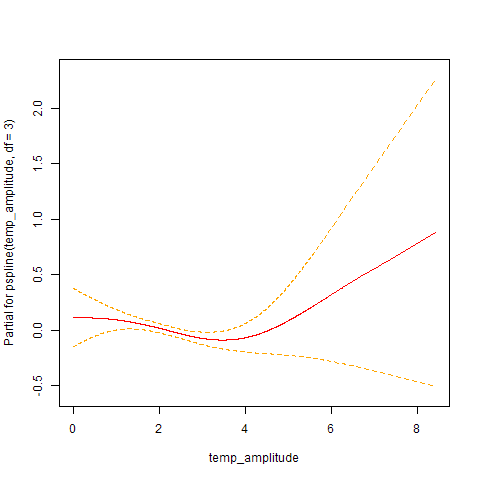


### Parkinson's disease


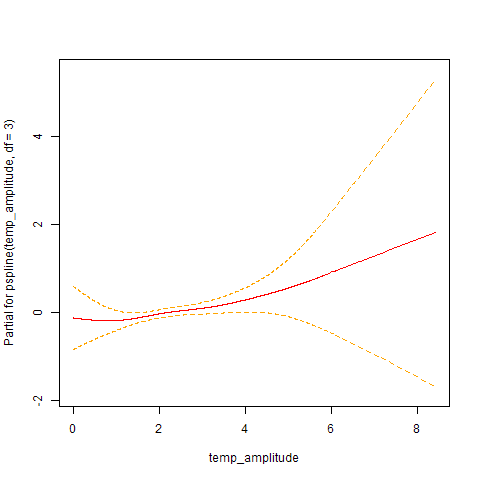


## Competing outcomes

Finally, we consider whether including death as a competing outcome influences the model. In our main manuscript, we consider death to be a censoring event, equivalent to the end of data collection for that individual. Here we instead simultaneously model both death and diagnosis of the phenotype as potential outcomes.

The results of running each of these models is displayed below. The table displays the p-value and effect size of the temperature amplitude effect. These can be compared to the main results in the manuscript for inconsistent results.

|  | p | logHR | logHR_se | std_logHR | std_logHR_se |
| --- | --- | --- | --- | --- | --- |
| Type 2 diabetes | 6.9123e-23 | -2.8963e-01 | 2.9406e-02 | -2.6372e-01 | 2.6775e-02 |
| Essential hypertension | 6.7476e-14 | -1.1531e-01 | 1.5389e-02 | -1.0499e-01 | 1.4012e-02 |
| Other chronic nonalcoholic liver disease | 3.7962e-11 | -3.5512e-01 | 5.3709e-02 | -3.2335e-01 | 4.8904e-02 |
| Renal failure | 2.6838e-06 | -1.2117e-01 | 2.5815e-02 | -1.1033e-01 | 2.3505e-02 |
| Diverticulosis | 1.2246e-08 | -9.9938e-02 | 1.7544e-02 | -9.0997e-02 | 1.5975e-02 |
| Pneumonia | 3.1531e-05 | -1.0759e-01 | 2.5851e-02 | -9.7968e-02 | 2.3538e-02 |
| Disorders of lipoid metabolism | 6.0682e-06 | -8.3728e-02 | 1.8508e-02 | -7.6238e-02 | 1.6852e-02 |
| Sleep disorders | 4.2162e-08 | -2.2422e-01 | 4.0905e-02 | -2.0416e-01 | 3.7246e-02 |
| Osteoarthrosis | 4.5994e-03 | -6.2649e-02 | 2.2107e-02 | -5.7044e-02 | 2.0130e-02 |
| Extrapyramidal disease and abnormal movement disorders | 2.1270e-05 | -2.8147e-01 | 6.6210e-02 | -2.5628e-01 | 6.0286e-02 |
| Anxiety disorders | 6.3087e-02 | -5.6573e-02 | 3.0439e-02 | -5.1512e-02 | 2.7716e-02 |
| Parkinson's disease | 2.5251e-02 | 1.5296e-01 | 6.8363e-02 | 1.3928e-01 | 6.2247e-02 |
