# Supplementary material for: Diurnal rhythms of wrist temperature are associated with future disease risk in the UK Biobank
Source: Nat Commun. 2023 Aug 24;14:5172. doi: 10.1038/s41467-023-40977-5 (PMC10449859; doi:10.1038/s41467-023-40977-5)
Supplement: Supplementary file 6 — Reporting Summary [file 41467_2023_40977_MOESM6_ESM.pdf]

nature portfolio

Corresponding author(s): Thomas Brooks and Carsten Skarke

Last updated by author(s): Jul 20, 2023

Reporting Summary

Nature Portfolio wishes to improve the reproducibility of the work that we publish. This form provides structure for consistency and transparency in reporting. For further information on Nature Portfolio policies, see our Editorial Policies and the Editorial Policy Checklist.

Statistics

For all statistical analyses, confirm that the following items are present in the figure legend, table legend, main text, or Methods section.

n/a	Confirmed	
☐	☒	The exact sample size (n) for each experimental group/condition, given as a discrete number and unit of measurement
☐	☒	A statement on whether measurements were taken from distinct samples or whether the same sample was measured repeatedly
☐	☒	The statistical test(s) used AND whether they are one- or two-sided<br>*Only common tests should be described solely by name; describe more complex techniques in the Methods section.*
☐	☒	A description of all covariates tested
☐	☒	A description of any assumptions or corrections, such as tests of normality and adjustment for multiple comparisons
☐	☒	A full description of the statistical parameters including central tendency (e.g. means) or other basic estimates (e.g. regression coefficient) AND variation (e.g. standard deviation) or associated estimates of uncertainty (e.g. confidence intervals)
☐	☒	For null hypothesis testing, the test statistic (e.g. F, t, r) with confidence intervals, effect sizes, degrees of freedom and P value noted<br>*Give P values as exact values whenever suitable.*
☒	☐	For Bayesian analysis, information on the choice of priors and Markov chain Monte Carlo settings
☒	☐	For hierarchical and complex designs, identification of the appropriate level for tests and full reporting of outcomes
☐	☒	Estimates of effect sizes (e.g. Cohen's d, Pearson's r), indicating how they were calculated

Our web collection on statistics for biologists contains articles on many of the points above.

Software and code

Policy information about availability of computer code

Data collection	The modified pipeline for processing the raw actigraphy data is available at https://github.com/tgbrooks/biobankAccelerometerAnalysis.
Data analysis	The source code for all statistical tests,and generated figures used in this analysis is available at GitHub https://github.com/tgbrooks/ukbb (DOI: 10.5281/zenodo.8169500 [https://doi.org/10.5281/zenodo.8169500]) and includes simulated data to test the statistics and analyses, with instructions in the README.md file. The analyses used Python 3.10.4 and R 4.2.2. Python package versions are specified in requirements.txt file. R packages timereg v2.0.5 and survival v3.4 were used.

For manuscripts utilizing custom algorithms or software that are central to the research but not yet described in published literature, software must be made available to editors and reviewers. We strongly encourage code deposition in a community repository (e.g. GitHub). See the Nature Portfolio guidelines for submitting code & software for further information.

Data

Policy information about availability of data

All manuscripts must include a data availability statement. This statement should provide the following information, where applicable:

- Accession codes, unique identifiers, or web links for publicly available datasets
- A description of any restrictions on data availability
- For clinical datasets or third party data, please ensure that the statement adheres to our policy

Data for this study was obtained from the UK Biobank Resource under Application Number 50398. The data is available to bona fide researchers for a fee from the

UK Biobank. Since this data is not widely available, a small, simulated dataset that mimics the characteristics of the real data is available along with our source code (see Code Availability section) and can be used to demonstrate or test our analyses. Source data are provided with this paper.

Research involving human participants, their data, or biological material

Policy information about studies with human participants or human data. See also policy information about sex, gender (identity/presentation), and sexual orientation and race, ethnicity and racism.

Reporting on sex and gender	All tests are performed and reported with both sexes combined and also separately by sex (Fig 3b and Table S1). Differences between sexes in results were small and discussed in the manuscript.
Reporting on race, ethnicity, or other socially relevant groupings	The study group was 97% white and no subgroup analyses by race or ethnicity were attempted.
Population characteristics	Population demographics are reported in Table S2. The study used the UK Biobank and exhibited an "healthy volunteer" bias compared to the overall biobank population. The population was 97% white and 56.3% female. The mean age was 62.8 (7.8 years SD). The mean BMI was 26.7 (4.5 SD).
Recruitment	Subjects were part of the UK Biobank and were recruited for participation in the activity monitoring study via email. Those who accepted were then mailed a wrist-worn actigraphy device which they wore for one week and mailed back afterwards. No restrictions were placed on the activities or travel of subjects during this time, other than needing to receive the device in the mail. Subjects were encouraged to wear the device even if their week was unusual for them. We observed a selection bias relative to the overall UK Biobank where participants were slightly whiter, more female, younger, healthier, and lower BMI. We report in Table S2 a demographics comparison to the overall UK Biobank population.
Ethics oversight	The study was performed according to the standards of the UK Biobank, under Application Number 50398.

Note that full information on the approval of the study protocol must also be provided in the manuscript.

Field-specific reporting

Please select the one below that is the best fit for your research. If you are not sure, read the appropriate sections before making your selection.

[x] Life sciences [ ] Behavioural & social sciences [ ] Ecological, evolutionary & environmental sciences

For a reference copy of the document with all sections, see nature.com/documents/nr-reporting-summary-flat.pdf

Life sciences study design

All studies must disclose on these points even when the disclosure is negative.

Sample size	A total of 91,462 individuals were included in the analysis. Each specific anlysis was conducted in subcohorts defined by the phenotype of interest with ranges of diagnosis events from 200 to 6,728 and from 67,107 to 91,134 participants without the diagnosis event. Power analysis was performed to determine that 200 events was sufficient for a 0.2 log HR effect size at 80% power; therefore, all phenotypes with at least 200 events were considered.
Data exclusions	Subjects were considered for inclusion if they had activity monitoring data. Subjects were excluded if they failed to meet any of three quality control measures according to existing pipelines for this dataset (bad calibration n=2826; insufficient wear time n=4,258; GMT/BST crossover n=4,279) or if they were missing any of the covariate information such as age (n=863). For each phenotype, subjects were excluded from the phenotype-specific analysis based on any existing diagnosis of the phenotype (or any closely related phenotype) prior to one year after the activity monitoring measurement. These criteria were performed according to the Phecode Map v1.2b defined exclusion criteria.
Replication	No independent replication data is available. To simulate independent replications, we divided the groups up according to observed clusters of activity montioring device IDs and computed the anlayses in two completely separate subsets of individuals according to their device ID. This confirmed in both subcohorts the reported findings of the study, except as noted in the manuscript (overall mortality was significantly associated with temperature rhythms in only one subcohort, possibly due to lower event counts).
Randomization	No randomization was performed. We controlled for covariates by inclusion in the Cox proportional hazards model. These factors were: sex (male/female, from NHS records or updated by participants), ethnicity (white/other, since 97% are white), smoking history (Prefer not to answer/Never/Previous/Current)), age at the time of actigraphy measure (40-55/55-60/60-65/65-70/70-80), BMI at assessment (continuous), college education (yes/no), Townsend deprivation index (continuous), alcohol consumption (Prefer not to answer/Daily or almost daily/Three or four times a week/Once or twice a week/One to three times a month/Special occasions only/Never)) and self-reported overall health (Do not know/Prefer not to answer/Excellent/Good/Fair/Poor), determined at initial assessment. All covariates were assessed at initial enrollment in the UKBB, which had a mean of 5.7 (2.8 - 8.7 min-max) years before actigraphy collection.
Blinding	No blinding was done. Blinding was impossible given the nature of the data (amplitudes are continuous variables that are not symmetric, so the analyzer will always know which subjects have low amplitudes).

Behavioural & social sciences study design

All studies must disclose on these points even when the disclosure is negative.

Study description	*Briefly describe the study type including whether data are quantitative, qualitative, or mixed-methods (e.g. qualitative cross-sectional, quantitative experimental, mixed-methods case study).*
Research sample	*State the research sample (e.g. Harvard university undergraduates, villagers in rural India) and provide relevant demographic information (e.g. age, sex) and indicate whether the sample is representative. Provide a rationale for the study sample chosen. For studies involving existing datasets, please describe the dataset and source.*
Sampling strategy	*Describe the sampling procedure (e.g. random, snowball, stratified, convenience). Describe the statistical methods that were used to predetermine sample size OR if no sample-size calculation was performed, describe how sample sizes were chosen and provide a rationale for why these sample sizes are sufficient. For qualitative data, please indicate whether data saturation was considered, and what criteria were used to decide that no further sampling was needed.*
Data collection	*Provide details about the data collection procedure, including the instruments or devices used to record the data (e.g. pen and paper, computer, eye tracker, video or audio equipment) whether anyone was present besides the participant(s) and the researcher, and whether the researcher was blind to experimental condition and/or the study hypothesis during data collection.*
Timing	*Indicate the start and stop dates of data collection. If there is a gap between collection periods, state the dates for each sample cohort.*
Data exclusions	*If no data were excluded from the analyses, state so OR if data were excluded, provide the exact number of exclusions and the rationale behind them, indicating whether exclusion criteria were pre-established.*
Non-participation	*State how many participants dropped out/declined participation and the reason(s) given OR provide response rate OR state that no participants dropped out/declined participation.*
Randomization	*If participants were not allocated into experimental groups, state so OR describe how participants were allocated to groups, and if allocation was not random, describe how covariates were controlled.*

Ecological, evolutionary & environmental sciences study design

All studies must disclose on these points even when the disclosure is negative.

Study description	*Briefly describe the study. For quantitative data include treatment factors and interactions, design structure (e.g. factorial, nested, hierarchical), nature and number of experimental units and replicates.*
Research sample	*Describe the research sample (e.g. a group of tagged Passer domesticus, all Stenocereus thurberi within Organ Pipe Cactus National Monument), and provide a rationale for the sample choice. When relevant, describe the organism taxa, source, sex, age range and any manipulations. State what population the sample is meant to represent when applicable. For studies involving existing datasets, describe the data and its source.*
Sampling strategy	*Note the sampling procedure. Describe the statistical methods that were used to predetermine sample size OR if no sample-size calculation was performed, describe how sample sizes were chosen and provide a rationale for why these sample sizes are sufficient.*
Data collection	*Describe the data collection procedure, including who recorded the data and how.*
Timing and spatial scale	*Indicate the start and stop dates of data collection, noting the frequency and periodicity of sampling and providing a rationale for these choices. If there is a gap between collection periods, state the dates for each sample cohort. Specify the spatial scale from which the data are taken*
Data exclusions	*If no data were excluded from the analyses, state so OR if data were excluded, describe the exclusions and the rationale behind them, indicating whether exclusion criteria were pre-established.*
Reproducibility	*Describe the measures taken to verify the reproducibility of experimental findings. For each experiment, note whether any attempts to repeat the experiment failed OR state that all attempts to repeat the experiment were successful.*
Randomization	*Describe how samples/organisms/participants were allocated into groups. If allocation was not random, describe how covariates were controlled. If this is not relevant to your study, explain why.*
Blinding	*Describe the extent of blinding used during data acquisition and analysis. If blinding was not possible, describe why OR explain why blinding was not relevant to your study.*

Did the study involve field work? ☐ Yes ☐ No

Field work, collection and transport

Field conditions	*Describe the study conditions for field work, providing relevant parameters (e.g. temperature, rainfall).*
Location	*State the location of the sampling or experiment, providing relevant parameters (e.g. latitude and longitude, elevation, water depth).*
Access & import/export	*Describe the efforts you have made to access habitats and to collect and import/export your samples in a responsible manner and in compliance with local, national and international laws, noting any permits that were obtained (give the name of the issuing authority, the date of issue, and any identifying information).*
Disturbance	*Describe any disturbance caused by the study and how it was minimized.*

Reporting for specific materials, systems and methods

We require information from authors about some types of materials, experimental systems and methods used in many studies. Here, indicate whether each material, system or method listed is relevant to your study. If you are not sure if a list item applies to your research, read the appropriate section before selecting a response.

Materials & experimental systems

n/a	Involved in the study	
☒	☐	Antibodies
☒	☐	Eukaryotic cell lines
☒	☐	Palaeontology and archaeology
☒	☐	Animals and other organisms
☒	☐	Clinical data
☒	☐	Dual use research of concern
☒	☐	Plants

Methods

n/a	Involved in the study	
☒	☐	ChIP-seq
☒	☐	Flow cytometry
☒	☐	MRI-based neuroimaging
